# A diagnostic model for sepsis-induced acute lung injury using a consensus machine learning approach and its therapeutic implications

**DOI:** 10.1186/s12967-023-04499-4

**Published:** 2023-09-12

**Authors:** Yongxin Zheng, Jinping Wang, Zhaoyi Ling, Jiamei Zhang, Yuan Zeng, Ke Wang, Yu Zhang, Lingbo Nong, Ling Sang, Yonghao Xu, Xiaoqing Liu, Yimin Li, Yongbo Huang

**Affiliations:** 1 Department of Critical Care Medicine, The First Affiliated Hospital of Guangzhou Medical University, Guangzhou, 510120 China; 2 Guangzhou Institute of Respiratory Health, Guangzhou, 510120 China; 3 State Key Laboratory of Respiratory Diseases, Guangzhou, 510120 China; 4 Department of Cardiovascular Medicine, The First Affiliated Hospital of Guangzhou Medical University, Guangzhou, 510120 Guangdong, China

**Keywords:** Sepsis, Acute lung injury, Acute respiratory distress syndrome, Machine learning, Transcriptome

## Abstract

**Background:**

A significant proportion of septic patients with acute lung injury (ALI) are recognized late due to the absence of an efficient diagnostic test, leading to the postponed treatments and consequently higher mortality. Identifying diagnostic biomarkers may improve screening to identify septic patients at high risk of ALI earlier and provide the potential effective therapeutic drugs. Machine learning represents a powerful approach for making sense of complex gene expression data to find robust ALI diagnostic biomarkers.

**Methods:**

The datasets were obtained from GEO and ArrayExpress databases. Following quality control and normalization, the datasets (GSE66890, GSE10474 and GSE32707) were merged as the training set, and four machine learning feature selection methods (Elastic net, SVM, random forest and XGBoost) were applied to construct the diagnostic model. The other datasets were considered as the validation sets. To further evaluate the performance and predictive value of diagnostic model, nomogram, Decision Curve Analysis (DCA) and Clinical Impact Curve (CIC) were constructed. Finally, the potential small molecular compounds interacting with selected features were explored from the CTD database.

**Results:**

The results of GSEA showed that immune response and metabolism might play an important role in the pathogenesis of sepsis-induced ALI. Then, 52 genes were identified as putative biomarkers by consensus feature selection from all four methods. Among them, 5 genes (ARHGDIB, ALDH1A1, TACR3, TREM1 and PI3) were selected by all methods and used to predict ALI diagnosis with high accuracy. The external datasets (E-MTAB-5273 and E-MTAB-5274) demonstrated that the diagnostic model had great accuracy with AUC value of 0.725 and 0.833, respectively. In addition, the nomogram, DCA and CIC showed that the diagnostic model had great performance and predictive value. Finally, the small molecular compounds (Curcumin, Tretinoin, Acetaminophen, Estradiol and Dexamethasone) were screened as the potential therapeutic agents for sepsis-induced ALI.

**Conclusion:**

This consensus of multiple machine learning algorithms identified 5 genes that were able to distinguish ALI from septic patients. The diagnostic model could identify septic patients at high risk of ALI, and provide potential therapeutic targets for sepsis-induced ALI.

**Supplementary Information:**

The online version contains supplementary material available at 10.1186/s12967-023-04499-4.

## Introduction

Sepsis is a major public health concern which develops an abnormal host response to an infection, and is associated with the life-threatening organ dysfunction [1, 2]. Acute respiratory distress syndrome (ARDS), a common and fatal complication of sepsis, is characterized by the damage of alveolar-capillary membrane leading to lung edema and hypoxemia [3]. In a large international study, approximately 75% of patients with ARDS were caused by sepsis [4]. According to the US report, there are over 210,000 cases of sepsis-induced ARDS in the US annually [5]. Besides, septic patients with ARDS had a higher overall disease severity, poorer recovery from lung injury and higher mortality than non-sepsis-related ALI [6]. Despite the growing understanding of the mechanisms in sepsis-induced ARDS, we still remain incompletely understood of why only a fraction of septic patients will develop ARDS. Furthermore, ARDS will develop rapidly after initial insult, and no consensus has yet been reached regarding biomarkers that can be used to directly diagnose ARDS and assess lung injury. Thus, it is important to identify some diagnostic biomarkers for the diagnosis of ARDS.

Gene expression signatures have been an intense focus of studies in recent years. Numerous studies have indicated that gene expression signatures have great predictive value to identify septic patients with ARDS [7]. In one study, an 8-gene signature was found to be associated with acute lung injury (ALI), which could be used to distinguish ALI patients from septic patients [8]. Then, the expression of genes related to neutrophils was significantly increased in septic patients with ARDS rather than patients with sepsis alone [9]. The recent study had also found the distinguishing gene expression profiles in monocytes between patients with sepsis and patients with sepsis with ARDS [10]. Thus, the gene signatures from gene expression profiles might be a novel and accurate biomarkers to distinguish patients with ARDS. However, with a large number of gene signatures involving the pathophysiological process, identifying those relevant for diagnosis of ARDS can be computationally challenging.

Machine learning is an emerging field with huge resources to deal with large, complex and disparate data. It has progressively improved our ability to find relevant features in large and high-dimensional data from gene expression profiles [11]. Supervised machine learning has been used successfully to develop classifiers for disease diagnosis and identify the related biomarkers on the basis of the input features [12, 13]. However, it still lacks the research using machine learning to identify potential diagnostic biomarkers of sepsis-induced ALI. Here, we hypothesized that by integrating multiple machine learning algorithms, we could identify gene expression signatures for sepsis-induced ALI, which may serve as diagnostic tools. Moreover, the functional analysis of the diagnostic genes identified will provide insight into the pathogenesis mechanisms of ALI development and uncover druggable targets for its prevention. In this study, we systematically reviewed the available transcriptomic profiling datasets, and identified the gene biomarkers associated with the diagnosis of sepsis-induced ALI by using a consensus of four different supervised machine learning features selection techniques. Further insight into the role of biomarkers in the pathogenesis of sepsis-induced ALI and potential candidates for the therapeutic intervention were explored.

## Methods

### Data sources used for analysis

**Fig. 1:**
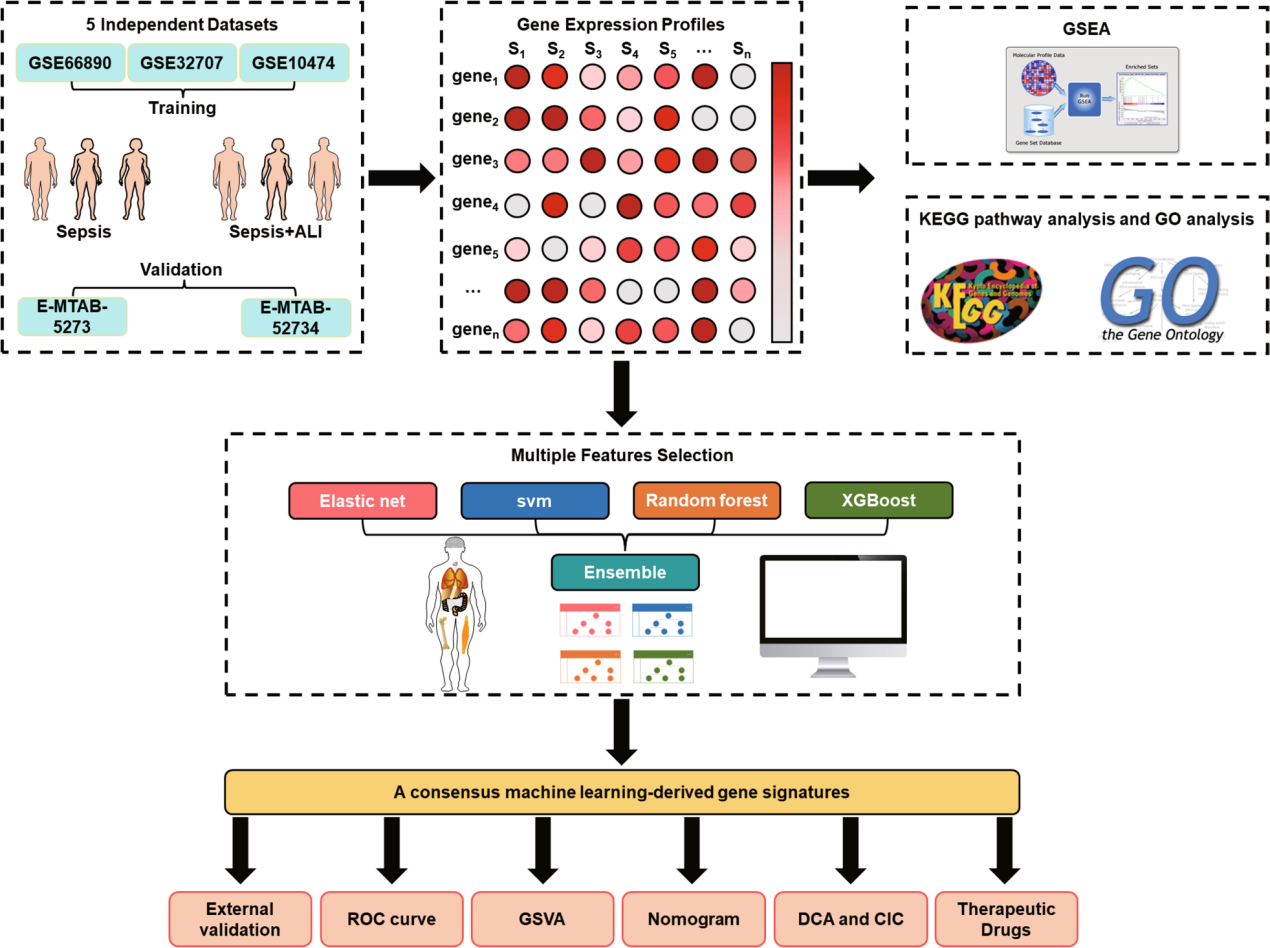
The overall flow of this study

The overall design of this study was shown in Fig. 1. We have retrospectively enrolled 5 datasets from Gene Expression Omnibus (GEO) (http://www.ncbi.nlm.nih.gov/geo) and ArrayExpress (https://www.ebi.ac.uk/arrayexpress/) database. Datasets between 2009 and 2020 containing transcriptomic profiling in Homo sapiens were potentially eligible. Datasets were excluded following the criteria: (1) the datasets included pediatric patients; (2) not measuring RNA; (3) patient’s samples were obtained after the admission over 48 h; (4) focusing on special pathogens such as *Staphylococcus aureus* and *Pseudomonas aeruginosa*; (5) not focusing on sepsis, sepsis-associated lung injury or sepsis-associated pneumonia. Additional datasets could be added by manual search of the references of included studies. The detailed baseline characteristics was summarized in Additional file 1: Table S1. Among these datasets, GSE66890, GSE10474 and GSE32707 were utilized to develop the diagnostic model. Then, E-MTAB-5273 and E-MTAB-5274 from the ArrayExpress database were applied to evaluate the performance of the diagnostic model in distinguishing sepsis-induced ALI patients.

### Data preprocessing and identification of differentially expressed genes (DEGs)

The datasets were downloaded from GEO and ArrayExpress databases, and the probe expression matrix was converted to gene expression based on the platform annotation file. The expression matrix was further normalized by robust multichip average (RMA). Genes were then filtered, keeping only those expressed in at least 10% of arrays. In cases where datasets had missing values, Multiple Imputation were conducted by using the weighted average from k-nearest neighbors (KNN) to handle the missing values. Then, the datasets GSE66890, GSE10474 and GSE32707 were merged by using the “comBat” function in the sva package to remove the batch effect among the datasets. To evaluate the batch effect, we conducted the Principal Component Analysis (PCA) and t-Distributed Stochastic Neighbor Embedding (t-SNE) to investigate the data. The DEGs analysis between sepsis and sepsis-induced ALI was performed using the limma package. The thresholds of DEGs were |log fold change (FC)|> 0.2 and P value < 0.05. Then, the results were visualized in volcano plots and heatmap plots which were constructed by using the R packages ggplot and pheatmap. The guidelines of the transparent reporting of a multivariable prediction model for individual prognosis or diagnosis (TRIPOD) statement were followed (Additional file 1: Table S2).

### Pathway enrichment analysis

Based on the normalized gene expression matrix, the R package clusterProfiler was used to conduct the GSEA analysis. The pathway gene sets were downloaded from the molecular signature database (MSigDB). Normalized enrichment score (NES) and false discovery rate (FDR) were applied to quantify enrichment magnitude and statistical significance, respectively [14, 15].

### Multivariable DEGs selection and model building

To further conduct the multivariable DEGs selection, we firstly need to eliminate the high mean absolute correlation of DEGs by using a correlation matrix method. For each DEGs, the mean absolute correlation based on the pair-wise correlations was calculated. If a pair-wise correlation was > 0.5, the DEGs with greater absolute correlation was removed by using the caret package in R [16].

In multiple DEGs selection, the four independent feature selection methods were simultaneously conducted to screen candidate biomarkers. The intersection amongst the four machine learning algorithms were considered the significant features. In each method, parameters were tuned using stratified tenfold cross-validation (repeated 10 times) on the training set, and the cross-validation was also performed to overcome the imbalance of outcome variables. Then, we subsequently create predictive classification models by using a supervised machine learning method for binary classification, based on the selected features from machine learning algorithms. Elastic net linear regression could be used to select the relevant DEGs on binomial logistic regression using glmnet package in R [17]. We chose the regularization parameter, λ, using tenfold cross-validation with binomial deviance as the criterion. A probability threshold of > 0.5 was used to determine whether septic patients with ALI or not. SVM is a supervised learning manner to select relevant characteristics and remove redundant characteristics using e1071 R package. Based on the best parameters, we chose Polynomial Kernel of svm to screen features. Boruta is a feature selection random forest wrapper algorithm used to obtain the relevant variables. We performed 300 items of the random forest normalized permutation importance function to attribute importance by using Boruta package. Then, we constructed the random forest model with the DEGs selected by Boruta using randomForest package [16]. XGBoost is a very effective method in in a range of classification problems. It is an extreme gradient boosting method which can rank features from most to least important by using XGBoost package in R [18]. The final parameters selected can be seen in Additional file 1: Table S3. Features contributing to more than 1% improvement in accuracy to the branch were considered importance. However, few algorithms possessed the ability to perfectly perform feature selection. Thus, we constructed an ensemble supervised machine learning model based on the ‘stacking’ method, which refers to fitting multiple machine learning models on the same dataset and using secondary modeling to learn how to best combine their predictions [19]. The above supervised machine learning algorithms were combined to generate a consensus model. An ensemble of predictions from each model were generated by averaging the predicted probabilities from each individual supervised machine learning algorithm (Additional file 1: Figure S1). Models with highest area under the receiver operating curve (AUROC) in cross-validation were selected as the optimal model.

### Multivariable classifier performance assessment and validation

To further evaluate the performance of the diagnostic model, the AUCs of all methods above were calculated using average of the cross validation across the whole dataset. Then, we also assessed the accuracy of diagnostic model through external validation. The two datasets (E-MTAB-5273 and E-MTAB-5274) from ArrayExpress database were applied to perform the verification, and the AUCs were also calculated. To further compare classifiers, we also looked at the performance of each supervised machine learning algorithm by using the evaluation metrics.

### Functional analysis of diagnostic features

To further explore why diagnostic genes contribute to the development of ALI, we defined the top 30% and bottom 30% of patients with diagnostic DEGs expression in the merged dataset as overexpression and low expression groups. Then, the differences and pathway activity change between groups were analyzed by gene set variation analysis (GSVA) [20].

### Nomogram, decision curve analysis (DCA) and clinical impact curve (CIC) of predictive model

Nomogram is a graphical tool that is designed to approximate complicate calculation quickly [21]. The selected gene signatures in diagnostic model were included to construct a nomogram model using rms package, which was established to predict the occurrence of ALI in septic patients. To validate the performance of nomogram, the concordance index (C-index) was calculated to assess the discrimination by a bootstrap method with 1000 resamples. Then, the calibration curve was plotted to observe the nomogram prediction probabilities against the observed rates. DCA curves are widely used to measure clinical utility of a specific model by comprehensively considering the relative value of benefits and harms associated with the prediction model, which can overcome the limitations of both traditional statistical metrics [22]. CIC could visually show the overall net benefit of nomogram within the wide and practical ranges of threshold probabilities that might impact patient outcomes, which indicates that the diagnostic model possesses significant predictive value [23]. Thus, in this study, the DCA curves and CIC were used to evaluate the predictive value of diagnostic model by using rmda package.

### Drugs screened and docking

Based on the functional analysis of 5 selected features, we screened 5 protein-coding genes for targeted drugs. Drug selection criteria focused on the expression of selected features in sepsis-induced ALI patients. We used Autodock for molecular docking to find the interaction between small molecules compound and selected genes. First, we obtained the catalog of small molecules compound that interacting with selected genes from the CTD database (http://ctdbase.org/), followed by the structures of small molecules compound from PDB database (https://rcsb.org/). Next, we downloaded the biological macromolecular structures of selected features from Uniprot database (https://uniprot.org/). Finally, the automatic docking of biological macromolecules and small molecular compounds was performed according to the standard docking process. The interaction of small molecular compounds and biological macromolecules was determined by lowest binding energy. The PyMol was used to visualize the results.

### Statistical analysis

All data processing and analysis were conducted in R version 4.2.0. Correlation analysis between two continuous variables were analyzed by Spearman Rank correlation analysis. Nonparametric test was used to compare the difference between two groups. The ROC curve used to predict binary categorical variables was implemented via pROC package. P value < 0.05 was regarded as statistically significant. Error bar span 95% confidence intervals.

## Results

### Screening for DEGs and underlying biological mechanisms

**Fig. 2:**
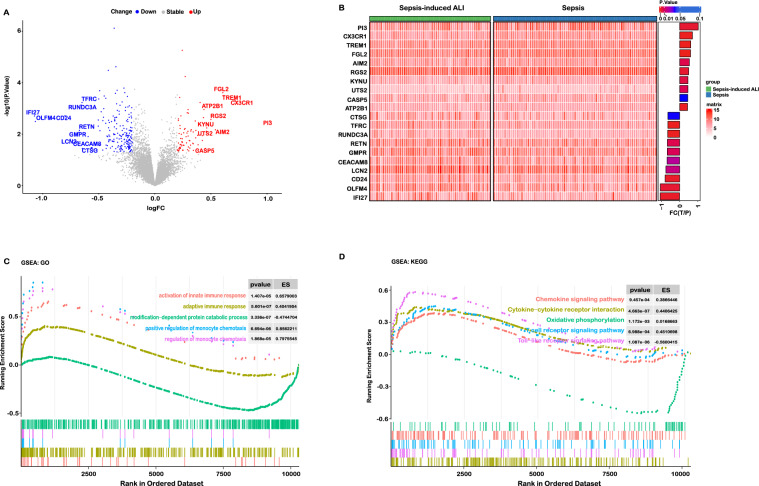
Different expression analysis and functional studies. **A**, **B** Volcano plot and heatmap showed the differentially expressed genes. **C**, **D** The biological functions were associated with the development of sepsis-induced ALI

According to the exclusion criteria, 3 microarray raw datasets containing a total of 79 cases of sepsis and 60 septic patients with ALI were included as the training set. The basic information of the included datasets is shown in Additional file 1: Table S2. Through gene expression profiles and PCA, we observed that there were baseline batch differences among the included datasets (Additional file 1: Figure S2A, B). To merge the datasets, the “combat” algorithm was applied to eliminate the batch effect which could increase the analysis power in the following analysis. After performing batch-correction approach, the batch differences were all eliminated (Additional file 1: Figure S2C, D). Among them, the sample (GSM812638, GSM812696, GSM812737, GSM812705, GSM812721) were removed because they could not be integrated. Initial t-SNE was also conducted to show some separation between groups (Additional file 1: Figure S2E, F). Then, DEGs were obtained by using limma package based on the P-value < 0.05 and |Log2FC|> 0.2. The different expression analysis revealed that there was 289 DEGs, including 76 upregulated and 193 downregulated genes (Fig. 2A, B).

To decipher the possible biological mechanisms underlying sepsis-induced ALI, we performed GSEA on 21,338 gene sets from MSigDB resource. The results showed that immune response and metabolism might play an important role in the development of ALI (Fig. 2C, D). Among them, the innate immune response, adaptive immune response and monocyte chemotaxis were significantly activated. Moreover, the pathways of chemokines secretion, Toll-like receptor and T cell receptor were also upregulated. To further explore the functional changes, we also conducted the functional enrichment analysis, including KEGG and GO analysis (Additional file 1: Figure S3). Based on the GO analysis, we found that the sepsis-induced ALI might be initiated by an inflammatory host response to a microbial pathogen (Additional file 1: Figure S3A). Then, mitochondrial plays an important role in the pathogenesis in sepsis-induced ALI. The mitochondrial biogenesis and other processes could be regulated by LPS (via TLR4 activation) involving the inflammatory and/or oxidative stress in tissues (Additional file 1: Figure S3B and D) [24]. Changes in alveolar epithelial and endothelial cells during sepsis-induced ALI include alterations in cell–cell junction formation, cell surface glycocalyx, and cell trauma or death (Additional file 1: Figure S3C, D). Thus, the results showed that aberrant host response to infection leads to the disruption of alveolar-capillary barrier, resulting in the development of lung injury. Dysregulated immune response was associated with the occurrence of sepsis-induced ALI, and monocytes might be the key immune cells contributing to the lung injury. Damage of endothelial and epithelial cells was essential for the progression of ALI.

### DEGs selected using supervised machine learning algorithms

**Fig. 3:**
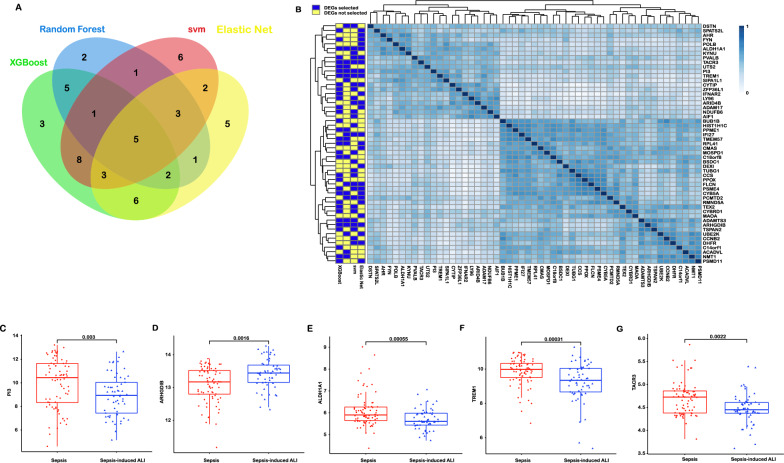
The DEGs selected by each machine learning methods. **A** Venn diagram showed the intersection of DEGs selected by four supervised machine learning approaches. **B** The expression correlation matrix among DEGs selected by all machine learning algorithms. **C**–**G** The expression levels of PI3, ARHGDIB, ALDH1A1, TREM1 and TACR3

In this study, we profiled the DEGs from 77 septic patients without ALI and 57 septic patients with ALI. Since several of the supervised machine learning approaches could not account for the multicollinearity, we removed the DEGs failing quality control and DEGs highly correlated with each other (Additional file 1: Figure S4). Then, remaining 70 genes selection was used to determine the DEGs most relevant to the diagnosis. Four different machine learning methods (Elastic net, svm, random forest and XGBoost) were performed to select DEGs and construct diagnostic model. Each features subsets selected by each method were different (Additional file 1: Figure S5A–D), and there were 5 genes overlapping in all (Fig. 3A, B). Basic on the importance of features, there were 27 genes were selected by Elastic net, 29 genes were selected by svm, 20 genes were selected by random forest and 33 genes were selected by XGBoost. The genes (ARHGDIB, ALDH1A1, TREM1, TACR3 and PI3) selected by all methods were further used to construct diagnostic model. The expression levels of selected features were showed in Fig. 3C–G. To ensure no individual features was driving the diagnostic model, a univariable analysis was conducted. For the DEGs selected by at least two methods, the expression levels of sepsis and sepsis-induced ALI were compared by using Wilcoxon signed-rank test, and controlled for multiple testing by using Benjamini Hochberg correction at 0.05. The centered expression values of DEGs selected by at least two methods were showed in Additional file 1: Figure S6A, B. 32 DEGs identified in the feature selection methods had an p-value < 0.05.

### Performance of diagnosis for sepsis-induced ALI using selected DEGs

**Fig. 4:**
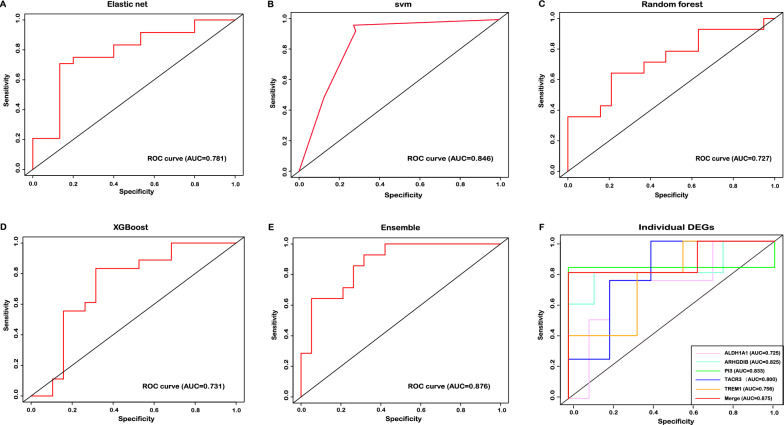
The performance of each feature selection method. **A** Elastic net utilizing 27 genes. **B** svm utilizing 29 genes. **C** Random forest utilizing 20 genes. **D** XGBoost utilizing 33 genes. **E** Ensemble approach utilizing 53 genes. **F** Average cross validated ROC for overlapping genes selected by four feature selection methods on the validation set

To compare the performance of each feature selection method, we evaluated how each model performed as a classifier on the validation set. As shown in Table 1, the svm model had the highest AUC (0.846) and accuracy (0.872). The random forest model had the poorest AUC (0.727) and accuracy (0.730) (Fig. 4A–D). As multivariable methods are known to select features with different accuracy, we conducted the ensemble learning algorithm using the DEGs selected by each model. The result showed that ensemble model had higher AUC (0.876) than svm model (Fig. 4E). Then, the number of DEGs selected by each model were also different, with the XGBoost model selecting the most genes and random forest model selecting the least genes (Table 1). Moreover, we further focused on the overlapping genes selected by four feature selection methods, and we evaluated the performance of individual overlapping genes in sepsis-induced ALI diagnosis. The result showed that PI3 had the best performance with the highest AUC (0.833). Then, the genes selected by all models were combined to construct the diagnostic model, and the model have great predictive value with higher AUC (0.875) (Fig. 4F). These results confirmed that the diagnostic model constructed by genes (ARHGDIB, ALDH1A1, TREM1, TACR3 and PI3) had perfect diagnostic efficiency. Thus, a clear association of selected features with sepsis-induced ALI diagnosis may warrant future investigation of specific genes for therapeutic intervention.

**Table 1:** Model performance of 4 classifiers in validation set

	Elastic net	Svm	Random forest	XGBoost	Ensemble
DEGs selected by model, n	27	29	20	33	53
Sensitivity	0.800	0.917	0.692	0.813	0.714
Specificity	0.792	0.852	0.750	0.714	0.789
Positive predictive value	0.706	0.733	0.643	0.684	0.714
Negative predictive value	0.864	0.958	0.789	0.833	0.789
Correct classification rate	0.795	0.872	0.730	0.757	0.758
AUC	0.781	0.846	0.727	0.731	0.876

### Validation of diagnosis for sepsis-induced ALI by using external datasets

**Fig. 5:**
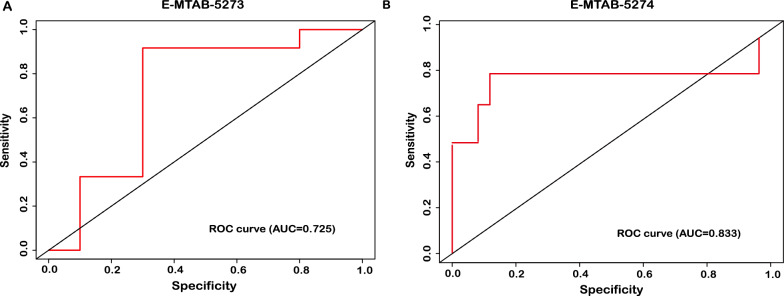
External validation of predictive performance in diagnostic model. **A** The ROC curve of E-MTAB-5273. **B** The ROC curve of E-MTAB-5274

To assess the predictive performance of diagnostic model, two datasets (E-MTAB-5273 and E-MTAB-5274) obtained from ArrayExpress database were considered as external validation. The overlapping genes selected by four supervised machine learning algorithms were used to conduct ROC analysis. The results showed that the AUC was 0.725 in E-MTAB-5273 (Fig. 5A) and 0.833 in E-MTAB-5274 (Fig. 5B). Thus, the results of external validation demonstrated that the diagnostic model constructed by 5 genes had excellent performance in sepsis-induced ALI.

### Visualization of the diagnostic model

**Fig. 6:**
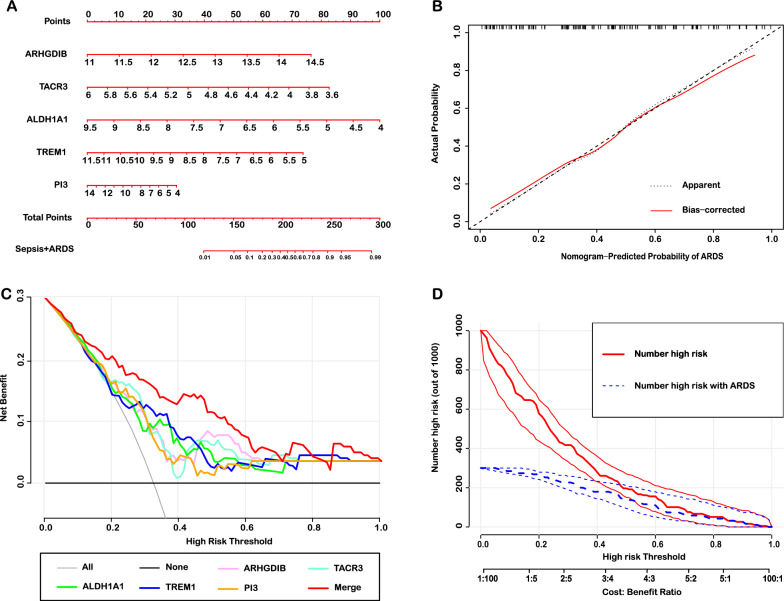
The nomogram, DCA and CIC of the diagnostic model. **A** Nomogram to evaluate the risk of the occurrence of sepsis-induced ALI. **B** Calibration curves of the nomogram prediction. **C** DCA curves of the nomogram prediction. **D** CIC of the nomogram prediction

For visualization of the diagnostic model, the risk nomogram that integrated 5 independent predictors for the incidence of sepsis-induced ALI (Fig. 6A). The calibration curves for incidence of sepsis-induced ALI indicated a high degree of overlap between the actual incidence rate and the incidence rate predicted by the nomogram (Fig. 6B), suggesting that nomogram has an excellent predictive value. Then, the decision curve analysis (DCA) for the diagnostic genes (ARHGDIB, ALDH1A1, TREM1, TACR3 and PI3) and that for the model with diagnostic genes integrated was presented in Fig. 6C. The DCA showed that if the threshold probability of a patients or doctor is > 10%, using the individual genes or diagnostic model to predict the occurrence of ALI adds more benefit than either diagnosis-all-patients scheme or the diagnosis-none scheme. Within this range, net benefit was comparable. The net benefit of integrated diagnostic model was superior than individual diagnostic genes (Fig. 6C). Based on the results of DCA, we further plotted the CIC to assess the clinical utility of the nomogram. The CIC visually showed that the nomogram with a superior overall net benefit within the wide and practical ranges of threshold probabilities and impacted the diagnosis, suggesting that the diagnostic model had an excellent predictive value (Fig. 6D). Besides, the CIC of the individual diagnostic genes were also showed the similar results (Additional file 1: Figure S7).

### Functional analysis and small molecular compound docking of diagnostic genes

**Fig. 7:**
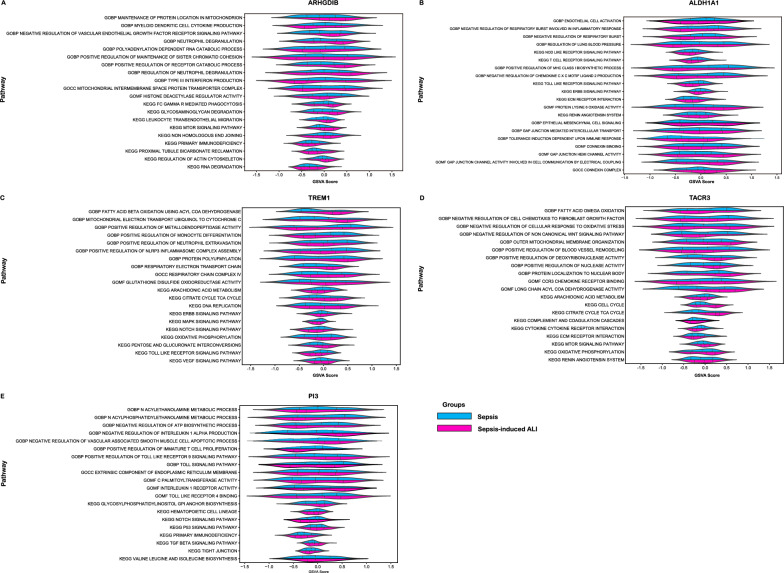
Functional analysis of diagnostic genes. **A**–**E** After grouping ARHGDIB (**A**), ALDH1A1 (**B**), TREM1 (**C**), TACR3 (**D**) and PI3 (**E**) at high and low levels, the enriched KEGG and GO pathways were scored for GSVA

A good biomarker is not only characterized by high specificity and sensitivity in diagnosing the disease but also yields valuable insights into the pathogenesis of the disease [25].Understanding the biological roles of specific diagnostic markers for ALI may help elucidate underlying mechanisms and lead to the identification of novel targets for therapeutic intervention. Thus we further explored the functional alteration of 5 diagnostic genes. Firstly, for ARHGDIB, it was significantly downregulated in sepsis-induced ALI. The results of GSVA after high expression showed that the activities of multiple immune response pathways, including neutrophils activation, were upregulated, indicating that ARHGDIB was involved in various immune and pathogen clearance in septic patients with ALI. Moreover, the upregulation of ARHGDIB in septic patients with ALI was also associated with negative regulation of vascular endothelial growth factor receptor signaling pathway, which might involve in the regulation of vascular permeability (Fig. 7A). Then, ALDH1A1 was expressed at a low level in sepsis-induced ALI. It was found that the upregulated ALDH1A1 could involve in the negative regulation of oxidative stress-related pathway such as respiratory burst. Furthermore, the endothelial cell activation pathway was also upregulated in septic patients with ALI, indicating that endothelial cell might synthesize and secrete some proteins and cytokines to promote the vascular permeability (Fig. 7B). As for TREM1, it is expressed on myeloid cells as a superimmunoglobulin receptor which could amplify the inflammatory response by interact with Toll-like receptor [26]. In this study, the septic patients without ALI had higher expression of TREM1, indicating that inflammatory response had an important role in the development of sepsis. Septic patients with ALI had lower expression of TREM1, followed with the mitochondrial dysfunction and downregulated biological metabolic pathways, such as oxidative phosphorylation, which might reduce energy production and further inhibit the vascular regeneration (Fig. 7C). Similarly, the decreased TACR3 in septic patients with ALI was also influenced the energy production (e.g., TCA cycle) and celluar replication (Fig. 7D), suggesting that TACR3 had an important role in tissue regeneration. PI3 was revealed a rapid decrease in ALI patients, which followed with the degrading extracellular matrix and decreased biosynthesis (Fig. 7E).

**Fig. 8:**
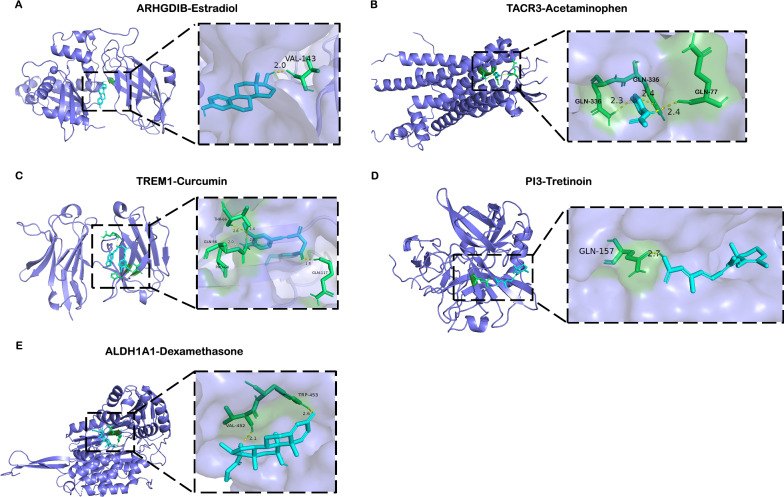
The docking results of diagnostic genes encoded proteins with small molecular compounds. **A** The docking result of ARHGDIB with Estradiol. **B** The docking result of TACR3 with Acetaminophen. **C** The docking result of TREM1 with Curcumin. **D** The docking result of PI3 with Tretinoin. **E** The docking result of ALDH1A1 with Dexamethasone

We next used the CTD database, drug toxicology studies and auto molecular docking to explore the drugs targeted to diagnostic genes. Firstly, we found that Estradiol could bind tightly with ARHGDIB and decrease the expression of ARHGDIB (Fig. 8A). Estradiol, as the naturally existing endogenous hormone in women, had been demonstrated that it could improve the pulmonary inflammation [27] and promote the proliferation of endothelial cells [28]. According to the GSVA results, we found that upregulated ARHGDIB was correlated with the increasing inflammation and inhibition of vascular endothelium regeneration. Thus, the results of molecular docking analysis indicated that Estradiol might ameliorate the lung injury by interacting with ARHGDIB with an optimal docking binding energy of -7.11(kcal/mol). Acetaminophen (also known as n-acetyl-p-aminophenol or APAP) was the famous analgesic and antipyretic agents, which could be used to block prostaglandin synthesis from arachidonic acid by inhibiting the enzymes cyclooxygenase (COX)-1 and -2 [29]. Moreover, Acetaminophen could also impact the activity of mitochondrial to affect the TCA cycle [30]. In our study, Acetaminophen could efficiently increase the expression of TACR3, which might enhance the production of biological energy by regulating the TCA cycle in mitochondrial (Fig. 8B). However, it still needs further study to prove the efficiency of Acetaminophen in treating sepsis-induced ALI. Curcumin is a polyphenolic compound derived from dietary spice turmeric which has several pharmacologic effects including anti-inflammatory, antioxidant, antiproliferative and antiangiogenic activities [31]. We found that Curcumin could blockage TREM1 by binding to TREM1 with high docking energy − 5.39 (kcal/mol), which might improve the inflammation and oxidative stress in septic patients (Fig. 8C). Tretinoin is a retinol (vitamin A) derivative which has been evaluated as a treatment for ARDS. In this study, Tretinoin could enhance the expression of PI3 with the high level of docking binding energy of up to − 6.71 (kcal/mol) (Fig. 8D). Dexamethasone, an approved corticosteroid medication, acting as an anti-inflammatory and immunosuppressant agent. It has been widely used to treat a variety of diseases, including ARDS and sepsis. In the results of molecular docking, Dexamethasone could bind tightly with ALDH1A1which will result in the decreased gene expression of ALDH1A1 (Fig. 8E).

## Discussion

ALI is a lethal clinical syndrome that commonly occurs in septic patients, but the pathogenesis is still unknown. The limitations of the current ALI diagnostic system hamper the capacity to early provide optimal clinical care to septic patients, as the clinical diagnosis of sepsis-induced ALI is primarily determined by PaO2/FiO2 and chest imaging, without regard to molecular biological characteristics [32, 33]. With the development of high-throughput sequencing technology and computational biology, numerous studies have proposed the predictive gene expression signatures based on various machine learning approaches. However, two questions should be considered that why a particular method should be used and which solution is the best one. The selection of algorithms by researchers may exist in the preference and bias. Thus, in this study, we integrated the gene expression profiles and performed a consensus machine learning algorithm to generate a consensus signature with high accuracy at identifying septic patients with ALI, as candidates for further investigation. We subsequently perform the external validation to assess the feasibility of diagnostic model in different centers, and the results suggested that the selected genes had a great predictive value with AUC (0.725 and 0.833). These data indicated that selected genes by combing different methods could reveal the diagnostic signatures and insights into regulators of disease.

The study has identified five gene signatures (ARHGDIB, ALDH1A1, TREM1, TACR3 and PI3) by several supervised machine learning algorithms (Additional file 1: Figure S8). ARHGDIB, the pivotal molecular in celluar signaling, is mainly expressed in hematopoietic tissues such as B- and T-lymphocyte cell line which was initially found to be act as the inhibitor of GDP dissociation from RhoA [34]. Previous studies had demonstrated that the upregulated ARHGDIB could promote the macrophages infiltration and increase the production of ROS by regulating the activity of NADPH oxidase in phagocytes [35, 36], indicating that the upregulated expression of ARHGDIB might aggravate the lung injury. Moreover, ARHGDIB could also inhibit the vascular endothelial cell migration and regulates vascular tone and other vascular functions [37]. The upregulated ARHGDIB could inhibit the expression of vascular endothelial growth factor (VEGF) which might suppress the regeneration of endothelial cells [38]. It has been found in this research that the overexpression of ARHGDIB in sepsis-induced ALI increases the activity of immune cells, and ARHGDIB had a significant negative correlation with the regeneration of vascular endothelial cell. It indicated that ARHGDIB promoted the development of ALI by affecting immune response and regulating activity of vascular, resulting in the damage of vascular endothelial cell and lung edema. It has been reported that the key role of ALDH1A1 is the oxidation of retinaldehyde to retinoic acid, forming transcriptional regulators critical for normal cell growth and differentiation [39]. Furthermore, the overexpression of ALDH1A1 is closely associated with system metabolism and inflammation. Studies have found that the high expression of ALDH1A1 predicts a poor prognosis because of dysregulated metabolism and inflammatory response [40, 41]. Interestingly, ALDH1A1 is low expression in septic patients with ALI. After the low expression of ALDH1A1 in the sepsis-induced ALI, it was found that the ability of immune tolerance was decreased, and the activities of related pathways of intercellular connectivity were also decreased, indicating that the low expression of ALDH1A1 might promote the damage of alveolar-endothelium barrier. TREM1, part of the immunoglobulin superfamily, was mainly expressed in neutrophils or monocytes/macrophages, when bound to ligand, stimulating release of proinflammatory cytokines (e.g., TNF-α and IL-1β). It is reported that the TREM1 can be used as a diagnostic and prognostic biomarker for sepsis, indicating the potential diagnostic value of TREM1 [42]. It is believed that the upregulated expression of TREM1 in response to infection will augment inflammatory response not only remove the pathogens but also aggravate the organs damage [42–44]. In this study, we found that the decreased expression of TREM1 in septic patients with ALI which might impair the clearance of pathogens. Besides, TREM1 is involved in the mitochondrial metabolism and energy production [45, 46]. The downregulating TREM1 will lead to mitochondrial metabolism disorder and reduce the energy production which affect the cell proliferation and repairment. Our research also found that the downregulating TACR3 was associated with the decreasing production of energy and enhanced oxidative stress. It is speculated that the redox imbalance and disturbed energy were induced by downregulating the expression of TACR3, leading to the development of ALI. PI3 is neutrophil serine proteinase inhibitor with a crucial role in preventing excessive tissue injury during inflammatory events. It has previously been identified as significantly downregulated in the acute stage of ARDS, in concordance with our findings [47]. The plasma PI3 levels could be used to early diagnosis ARDS, indicating that direct analysis of ARDS patient blood may provide valuable information [47]. Furthermore, the expression and polymorphisms in PI3 gene were significantly associated with ARDS risk which could be regarded as a prognostic marker [48, 49]. After injury-inducing, the epithelial will be repaired by secreting extracellular matrix to restore the epithelial barrier [50]. However, the downregulating of PI3 affected the secretion of extracellular matrix protein which might delay the tissue repair [47].These results suggest that the dysregulated immune response and enhanced oxidative stress might be the crucial initial mechanism to damage the alveolar-endothelium barrier, leading to increased permeability to liquid and protein across the lung endothelium, which then leads to oedema in the lung interstitium. Besides, mitochondrial dysfunction and bioenergetic dysfunction also largely contribute to the progression of sepsis-associated ALI. Thus, understanding the function of diagnostic genes will help to clarify the pathogenesis of sepsis-induced ALI and proposed the targeted therapy options.

Nowadays, reorientation of drug function is the novel strategy for disease treatment. With the ARDS mechanisms continued to reveal and treatment plans continued to refine, a variety of drugs were applied to treat ALI/ARDS. In COVID-19 associated ARDS, a lot of drugs were explored to treat COVID-19 patients even they were not applied to the treatment of lung diseases before [51, 52]. Therefore, according to this strategy, we performed targeted drug screening of diagnostic genes to propose a novel therapeutic approach for inhibiting the development of sepsis-associated ALI. As a small molecular compound, Estradiol could efficiently bind to and decrease ARHGDIB expression. Estrogen receptor are expressed in all immune cells which could regulate the cellular functions as transcriptional factor. Treatment with Estradiol will decrease the accumulation of immune cells (e.g., neutrophil and monocyte) and suppress the production of proinflammatory cytokines, which could improve the lung inflammation [53, 54]. However, excessive intake of Estrogen will result in the side effect such as vomiting, nausea and thrombosis [55]. Acetaminophen is one of the most popular analgesic and antipyretic agents, which showed an exceptional performance in increasing TACR3 expression. Previous studies have demonstrated that treating sepsis patients with Acetaminophen will reduce oxidative stress and inhibit the excessive innate immune response [56, 57], which is benefit for the tissue repair. The toxicity of Acetaminophen should be noticed that the overdose of Acetaminophen will lead to acute liver failure [58]. The herbal compounds Curcumin have been reported the beneficial effects in treating inflammatory diseases, neurological diseases, cardiovascular diseases, pulmonary disease, metabolic diseases, liver diseases, and cancers [59]. In sepsis-induced ALI, intranasal Curcumin could significantly reduce the expression of oxidative stress marker (e.g., nitric oxide (NO) and malondialdehyde (MDA)) and inflammatory cytokines (e.g., TNF-α). Besides, Curcumin also improves the lung permeability and reduce the capillary leakage [60]. Yuan et al. further demonstrated that curcumin exerts anti-inflammatory and anti-oxidant effects through regulation of TREM-1 gene activity, which is in line with our study [61]. Tretinoin (vitamin A derivative) was one of the compounds with upregulation of PI3 that exhibit high affinity docking binding energy. Tretinoin is a medicine with anti-inflammatory and immunomodulating properties for sepsis. Treatment with Tretinoin in sepsis will inhibit the activation of NF-κB and related target genes such as IL-6, MCP-1 and COX-2 [62]. Furthermore, Tretinoin also attenuated the fibroblast degradation of extracellular matrix, suggesting that Tretinoin could modify tissue injury and ameliorate the lung fibrosis [63]. Therefore, the interaction between Tretinoin and PI3 might improve the lung inflammation and fibrosis. Dexamethasone has been recognized as one of the most efficient anti-inflammatory medicines which was used in various inflammatory diseases. Early administration of Dexamethasone could reduce the overall mortality in ARDS patients [64]. Paradoxically, these hormones were given to patients with sepsis and pneumonia could not find the beneficial therapeutic efficacy [65, 66]. In our study, we found that Dexamethasone could increase the expression of ALDH1A1 in septic patients with ALI, which might prevent the lung inflammation and improve lung permeability. However, when administered through a systemic route, Dexamethasone can elicit severe side effects, such as hyperglycemia, hypertension, hydro-electrolytic disorders and peptic ulcers [67]. Thus, based on the drugs screening for targeting the five diagnostic genes, our study has proposed a novel targeted therapy strategy with a combination of multiple drugs, which might prevent the development of sepsis-induced ALI brought by the five diagnostic genes and improve the prognosis of patients. However, of the gene-targeted drugs selected in this study, the primary goal is regulating the mRNA expression of targeted genes. Further research is needed to explore the novel biomaterials to deliver drugs to targeted genes.

The novelty of this study lies in the integration of multiple machine learning algorithms to construct a consensus model for distinguishing septic patients with ALI or not. We firstly used the correlation matrix to eliminate the multicollinearity and performed multiple supervised machine learning approaches for constructing diagnostic model. Then, we further used the external datasets to validate the accuracy in diagnostic model. Further investigation discussing gene function and targeted drugs is also novel in this research. However, there still have some limitations in this study. Firstly, although we have performed a batch correction for the several datasets, the essential bath effect still exists. Future integration studies could begin with sequenced documents to ensure consistency and accuracy. Second, many genes were excluded during the merging of datasets and eliminating multicollinearity, resulting in the loss of some important genes. However, to validate the model in independent datasets, we must ensure that genes used for model construction were available in testing sets. Third, some clinical and molecular traits were not adequately provided in public datasets, which limited the study to further reveal the potential associations between diagnostic genes and some traits. Finally, while our study provides a framework for the early diagnosis through the assessment of specific genes, the results are still in the analytical and speculative stage without experiments validation, and we recognize that the process of assessing these diagnostic genes by microarray may be time-consuming. However, utilizing real-time PCR to assess the expression of these 5 genes offers as a quick and relatively straightforward method for early recognition of sepsis-associated ALI. Thus, nanogram of five genes measured by real-time PCR may represent a promising step towards meeting the urgent diagnostic needs in the context of rapidly progressing conditions like sepsis associated ALI. Future research may further refine this method and explore its integration with clinical practice to enhance its usability and effectiveness. Besides, the combined therapeutic value of the five targeted drugs at cellular and animal level will also need to further study. Based on the diagnostic model, we hope to establish a shared platform to aid in clinical diagnosis and treatment in sepsis-induced ALI.

## Conclusion

Our study using four supervised machine learning feature selection approaches identified a five gene signatures for sepsis-induced ALI from patient whole blood. These diagnostic genes could be used to construct a diagnostic model with great predictive value, which could be effectively distinguished septic patients with ALI or not. The selected signatures revealed the disease mechanisms that damage of alveolar-endothelium barrier and dysfunctions of mitochondrial metabolism may be the crucial mechanisms for the development of sepsis-associated ALI. Lastly, diagnostic genes may be the future putative drug targets, and drugs screened for the presence of diagnostic genes, leading to new sight for targeted therapy.

### Supplementary Information

**Additional file 1: Figure S1.** The architecture of stacking model involves multiple base models (Elastic Net, svm, Random Forest and XGBoost), and a meta-model (stacking ensemble model) that combines the predictions in the base models. The meta-model is trained on the predictions made by base models on out-of-sample data. In this study, the training datasets were prepared for the meta-model is via 10-fold cross-validation of base models, where the out-of-fold predictions are used as the basis for the training dataset for the meta-model. Multiple base-models are often complex and diverse, using a diverse range of models (Elastic Net, svm, Random Forest and XGBoost) that make different assumptions about the prediction. The meta-model is often simple which provide a smooth interpretation of the predictions made by the base models. Thus, linear models were used as the meta-model, and the predictions made is a weighted average of the predictions made by the base models. **Figure S2.** Data processing. **A**, **B** PCA analysis and Box plot of expression profiles before batch effect correction. **C**, **D** PCA analysis and Box plot of expression profiles after batch effect correction. **E** t-SNE plot of subjects before batch correction with Combat. **F** t-SNE plot of subjects after batch correction with Combat. **Figure S3.** The functional analysis for sepsis-induced ALI. **A** Biological process. **B** Cell component. **C** Molecular function. **D** KEGG pathway enrichment analysis. **Figure S4. **Correlation plot of genes remaining after filtering out those with high correlation (Spearman’s >0.5). **Figure S5.** The DEGs selected by each machine learning methods. **A**–**D** Elastic net, svm, random forest and XGBoost were conducted to select features and sort by importance. **Figure S6. **The expression levels of genes selected by at least two methods. **A** The expression of selected genes in sepsis and sepsis-induced ALI. **B** Heatmap showed the selected genes expression in sepsis and sepsis-induced ALI. **Figure S7**. Individual diagnostic genes CIC used to assess the performance of nomogram. **A**–**D** The CIC of ARHGDIB, ALDH1A1, TACR3, TREM1 and PI3. **Figure S8**. Potential mechanisms of diagnostic genes contributing to the development of ALI/ARDS. **Table S1**. Basic information of the datasets included in this study. **Table S2.** Tripod Checklist. **Table S3**: Parameters used to optimise an XGBoost classifier for sepsis-induced using genes. **a** the range of each parameter tuned, **b** the optimal parameter for the initial xgboost model, **c** the final parameter value used for an xgboost model trained on a reduced number of genes.

## Data Availability

All data used to support the findings of this study are included within the article. The datasets used and analyzed during the current study are available from GEO (http://www.ncbi.nlm.nih.gov/geo). The analyzed datasets generated during the study are available from the corresponding author upon reasonable request.

## Abbreviations

ARDS: Acute respiratory distress syndrome
ALI: Acute lung injury
GEO: Gene Expression Omnibus
RMA: Robust multichip average
PCA: Principal Component Analysis
t-SNE: T-Distributed Stochastic Neighbor Embedding
DCA: Decision curve analysis
CIC: Clinical impact curve
DEGs: Differentially expressed genes

## References

[1] Singer M, Deutschman CS, Seymour CW, Shankar-Hari M, Annane D, Bauer M, Bellomo R, Bernard GR, Chiche JD, Coopersmith CM (2016). The third international consensus definitions for sepsis and septic shock (Sepsis-3). JAMA.

[2] Mayr FB, Yende S, Angus DC (2014). Epidemiology of severe sepsis. Virulence.

[3] Gorman EA, O’Kane CM, McAuley DF (2022). Acute respiratory distress syndrome in adults: diagnosis, outcomes, long-term sequelae, and management. Lancet.

[4] Bellani G, Laffey JG, Pham T, Fan E, Brochard L, Esteban A, Gattinoni L, van Haren F, Larsson A, McAuley DF (2016). Epidemiology, patterns of care, and mortality for patients with acute respiratory distress syndrome in intensive care units in 50 Countries. JAMA.

[5] Clock UaWP. US and world population estimates (2018) United States Census Bureau website. https://www.census.gov/popclock/?intcmp=w_200x402.

[6] Sheu CC, Gong MN, Zhai R, Chen F, Bajwa EK, Clardy PF, Gallagher DC, Thompson BT, Christiani DC (2010). Clinical characteristics and outcomes of sepsis-related vs non-sepsis-related ARDS. Chest.

[7] Wang YM, Qi X, Gong FC, Chen Y, Yang ZT, Mao EQ, Chen EZ (2020). Protective and predictive role of Mucin1 in sepsis-induced ALI/ARDS. Int Immunopharmacol.

[8] Howrylak JA, Dolinay T, Lucht L, Wang Z, Christiani DC, Sethi JM, Xing EP, Donahoe MP, Choi AM (2009). Discovery of the gene signature for acute lung injury in patients with sepsis. Physiol Genomics.

[9] Demaret J, Venet F, Friggeri A, Cazalis MA, Plassais J, Jallades L, Malcus C, Poitevin-Later F, Textoris J, Lepape A, Monneret G (2015). Marked alterations of neutrophil functions during sepsis-induced immunosuppression. J Leukoc Biol.

[10] Jiang Y, Rosborough BR, Chen J, Das S, Kitsios GD, McVerry BJ, Mallampalli RK, Lee JS, Ray A, Chen W, Ray P (2020). Single cell RNA sequencing identifies an early monocyte gene signature in acute respiratory distress syndrome. JCI Insight.

[11] Toh TS, Dondelinger F, Wang D (2019). Looking beyond the hype: applied AI and machine learning in translational medicine. EBioMedicine.

[12] Hira ZM, Gillies DF (2015). A review of feature selection and feature extraction methods applied on microarray data. Adv Bioinform.

[13] Schrider DR, Kern AD (2018). Supervised machine learning for population genetics: a new paradigm. Trends Genet.

[14] Subramanian A, Tamayo P, Mootha VK, Mukherjee S, Ebert BL, Gillette MA, Paulovich A, Pomeroy SL, Golub TR, Lander ES, Mesirov JP (2005). Gene set enrichment analysis: a knowledge-based approach for interpreting genome-wide expression profiles. Proc Natl Acad Sci U S A.

[15] Eraso-Pichot A, Braso-Vives M, Golbano A, Menacho C, Claro E, Galea E, Masgrau R (2018). GSEA of mouse and human mitochondriomes reveals fatty acid oxidation in astrocytes. Glia.

[16] Errington N, Iremonger J, Pickworth JA, Kariotis S, Rhodes CJ, Rothman AM, Condliffe R, Elliot CA, Kiely DG, Howard LS (2021). A diagnostic miRNA signature for pulmonary arterial hypertension using a consensus machine learning approach. EBioMedicine.

[17] Ball KD, Erman B, Dill KA (2002). The elastic net algorithm and protein structure prediction. J Comput Chem.

[18] Chen T, Guestrin C: XGBoost. A scalable tree boosting System. In: Proceedings of the 22nd ACM SIGKDD international conference on knowledge discovery and data mining. 2016;785–794.

[19] Zhang L, Wang Z, Zhou Z, Li S, Huang T, Yin H, Lyu J (2022). Developing an ensemble machine learning model for early prediction of sepsis-associated acute kidney injury. iScience.

[20] Pang J, Yu Q, Chen Y, Yuan H, Sheng M, Tang W (2022). Integrating Single-cell RNA-seq to construct a Neutrophil prognostic model for predicting immune responses in non-small cell lung cancer. J Transl Med.

[21] Park SY (2018). Nomogram: an analogue tool to deliver digital knowledge. J Thorac Cardiovasc Surg.

[22] Vickers AJ, van Calster B, Steyerberg EW (2019). A simple, step-by-step guide to interpreting decision curve analysis. Diagn Progn Res.

[23] Hou N, Li M, He L, Xie B, Wang L, Zhang R, Yu Y, Sun X, Pan Z, Wang K (2020). Predicting 30-days mortality for MIMIC-III patients with sepsis-3: a machine learning approach using XGboost. J Transl Med.

[24] Suliman HB, Piantadosi CA (2014). Mitochondrial biogenesis: regulation by endogenous gases during inflammation and organ stress. Curr Pharm Des.

[25] Shen Z (2013). Cancer biomarkers and targeted therapies. Cell Biosci.

[26] Yuan Z, Syed M, Panchal D, Joo M, Bedi C, Lim S, Onyuksel H, Rubinstein I, Colonna M, Sadikot RT (2016). TREM-1-accentuated lung injury via miR-155 is inhibited by LP17 nanomedicine. Am J Physiol Lung Cell Mol Physiol.

[27] da Anunciacao LF, Sousa MN, Vidal-Dos-Santos M, Armstrong-Jr R, Moreira LFP, Correia CJ, Breithaupt-Faloppa AC (2022). Modulatory effects of 17beta-estradiol on acute lung inflammation after total occlusion of the descending aorta in male rats. Int Immunopharmacol.

[28] Filipe C, Lam Shang Leen L, Brouchet L, Billon A, Benouaich V, Fontaine V, Gourdy P, Lenfant F, Arnal JF, Gadeau AP, Laurell H (2008). Estradiol accelerates endothelial healing through the retrograde commitment of uninjured endothelium. Am J Physiol Heart Circ Physiol.

[29] Aminoshariae A, Khan A (2015). Acetaminophen: old drug, new issues. J Endod.

[30] Behrends V, Giskeodegard GF, Bravo-Santano N, Letek M, Keun HC (2019). Acetaminophen cytotoxicity in HepG2 cells is associated with a decoupling of glycolysis from the TCA cycle, loss of NADPH production, and suppression of anabolism. Arch Toxicol.

[31] Anand P, Kunnumakkara AB, Newman RA, Aggarwal BB (2007). Bioavailability of curcumin: problems and promises. Mol Pharm.

[32] Force ADT, Ranieri VM, Rubenfeld GD, Thompson BT, Ferguson ND, Caldwell E, Fan E, Camporota L, Slutsky AS (2012). Acute respiratory distress syndrome: the Berlin definition. JAMA.

[33] Antcliffe DB, Burnham KL, Al-Beidh F, Santhakumaran S, Brett SJ, Hinds CJ, Ashby D, Knight JC, Gordon AC (2019). Transcriptomic signatures in sepsis and a differential response to steroids. from the Vanish randomized trial. Am J Respir Crit Care Med.

[34] Olofsson B (1999). Rho guanine dissociation inhibitors: pivotal molecules in cellular signalling. Cell Signal.

[35] Said N, Sanchez-Carbayo M, Smith SC, Theodorescu D (2012). RhoGDI2 suppresses lung metastasis in mice by reducing tumor versican expression and macrophage infiltration. J Clin Invest.

[36] Geng J, Sun X, Wang P, Zhang S, Wang X, Wu H, Hong L, Xie C, Li X, Zhao H (2015). Kinases Mst1 and Mst2 positively regulate phagocytic induction of reactive oxygen species and bactericidal activity. Nat Immunol.

[37] Nagar H, Kim S, Lee I, Choi SJ, Piao S, Jeon BH, Shong M, Kim CS (2021). CRIF1 deficiency suppresses endothelial cell migration via upregulation of RhoGDI2. PLoS ONE.

[38] Xia B, Wang J (2019). Adenosine inhibits ovarian cancer growth through regulating RhoGDI2 protein expression. Drug Des Devel Ther.

[39] Li B, Yang K, Liang D, Jiang C, Ma Z (2021). Discovery and development of selective aldehyde dehydrogenase 1A1 (ALDH1A1) inhibitors. Eur J Med Chem.

[40] Kiefer FW, Orasanu G, Nallamshetty S, Brown JD, Wang H, Luger P, Qi NR, Burant CF, Duester G, Plutzky J (2012). Retinaldehyde dehydrogenase 1 coordinates hepatic gluconeogenesis and lipid metabolism. Endocrinology.

[41] Sanders TJ, McCarthy NE, Giles EM, Davidson KL, Haltalli ML, Hazell S, Lindsay JO, Stagg AJ (2014). Increased production of retinoic acid by intestinal macrophages contributes to their inflammatory phenotype in patients with Crohn’s disease. Gastroenterology.

[42] Ventetuolo CE, Levy MM (2008). Biomarkers: diagnosis and risk assessment in sepsis. Clin Chest Med.

[43] Gibot S, Cravoisy A, Levy B, Bene MC, Faure G, Bollaert PE (2004). Soluble triggering receptor expressed on myeloid cells and the diagnosis of pneumonia. N Engl J Med.

[44] Gibot S, Cravoisy A, Kolopp-Sarda MN, Bene MC, Faure G, Bollaert PE, Levy B (2005). Time-course of sTREM (soluble triggering receptor expressed on myeloid cells)-1, procalcitonin, and C-reactive protein plasma concentrations during sepsis. Crit Care Med.

[45] Tammaro A, Scantlebery AML, Rampanelli E, Borrelli C, Claessen N, Butter LM, Soriani A, Colonna M, Leemans JC, Dessing MC, Florquin S (2019). TREM1/3 deficiency impairs tissue repair after acute kidney injury and mitochondrial metabolic flexibility in tubular epithelial cells. Front Immunol.

[46] Liu C, Liu R, Cao Z, Guo Q, Huang H, Liu L, Xiao Y, Duan C, Ma R (2022). Identification of MMP9 as a novel biomarker to mitochondrial metabolism disorder and oxidative stress in calcific aortic valve stenosis. Oxid Med Cell Longev.

[47] Wang Z, Beach D, Su L, Zhai R, Christiani DC (2008). A genome-wide expression analysis in blood identifies pre-elafin as a biomarker in ARDS. Am J Respir Cell Mol Biol.

[48] Tejera P, Wang Z, Zhai R, Su L, Sheu CC, Taylor DM, Chen F, Gong MN, Thompson BT, Christiani DC (2009). Genetic polymorphisms of peptidase inhibitor 3 (elafin) are associated with acute respiratory distress syndrome. Am J Respir Cell Mol Biol.

[49] Wang T, Zhu Z, Liu Z, Yi L, Yang Z, Bian W, Chen W, Wang S, Li G, Li A (2017). Plasma neutrophil elastase and elafin as prognostic biomarker for acute respiratory distress syndrome: a multicenter survival and longitudinal prospective observation study. Shock.

[50] Matthay MA, Zemans RL, Zimmerman GA, Arabi YM, Beitler JR, Mercat A, Herridge M, Randolph AG, Calfee CS (2019). Acute respiratory distress syndrome. Nat Rev Dis Primers.

[51] Yin J, Li C, Ye C, Ruan Z, Liang Y, Li Y, Wu J, Luo Z (2022). Advances in the development of therapeutic strategies against COVID-19 and perspectives in the drug design for emerging SARS-CoV-2 variants. Comput Struct Biotechnol J.

[52] Asselah T, Durantel D, Pasmant E, Lau G, Schinazi RF (2021). COVID-19: discovery, diagnostics and drug development. J Hepatol.

[53] Speyer CL, Rancilio NJ, McClintock SD, Crawford JD, Gao H, Sarma JV, Ward PA (2005). Regulatory effects of estrogen on acute lung inflammation in mice. Am J Physiol Cell Physiol.

[54] Vermillion MS, Ursin RL, Attreed SE, Klein SL (2018). Estriol reduces pulmonary immune cell recruitment and inflammation to protect female mice from severe influenza. Endocrinology.

[55] Sj R (2021). The immune microenvironment in human papilloma virus-induced cervical lesions-evidence for estrogen as an immunomodulator. Front Cell Infect Microbiol.

[56] Husain AA, Martin GS (2015). What is old is new again: acetaminophen as a novel approach to treating sepsis. Crit Care Med.

[57] Janz DR, Bastarache JA, Rice TW, Bernard GR, Warren MA, Wickersham N, Sills G, Oates JA, Roberts LJ, Ware LB (2015). Randomized, placebo-controlled trial of acetaminophen for the reduction of oxidative injury in severe sepsis: the acetaminophen for the reduction of oxidative injury in severe sepsis trial. Crit Care Med.

[58] Ghanem CI, Pérez MJ, Manautou JE, Mottino AD (2016). Acetaminophen from liver to brain: new insights into drug pharmacological action and toxicity. Pharmacol Res.

[59] Jäger R, Lowery RP, Calvanese AV, Joy JM, Purpura M, Wilson JM (2014). Comparative absorption of curcumin formulations. Nutr J.

[60] Kumari A, Tyagi N, Dash D, Singh R (2015). Intranasal curcumin ameliorates lipopolysaccharide-induced acute lung injury in mice. Inflammation.

[61] Yuan Z, Syed MA, Panchal D, Rogers D, Joo M, Sadikot RT (2012). Curcumin mediated epigenetic modulation inhibits TREM-1 expression in response to lipopolysaccharide. Int J Biochem Cell Biol.

[62] Austenaa LM, Carlsen H, Hollung K, Blomhoff HK, Blomhoff R (2009). Retinoic acid dampens LPS-induced NF-kappaB activity: results from human monoblasts and in vivo imaging of NF-kappaB reporter mice. J Nutr Biochem.

[63] Zhu YK, Liu X, Ertl RF, Kohyama T, Wen FQ, Wang H, Spurzem JR, Romberger DJ, Rennard SI (2001). Retinoic acid attenuates cytokine-driven fibroblast degradation of extracellular matrix in three-dimensional culture. Am J Respir Cell Mol Biol.

[64] Villar J, Ferrando C, Martinez D, Ambros A, Munoz T, Soler JA, Aguilar G, Alba F, Gonzalez-Higueras E, Conesa LA (2020). Dexamethasone treatment for the acute respiratory distress syndrome: a multicentre, randomised controlled trial. Lancet Respir Med.

[65] Annane D, Sebille V, Bellissant E (2006). Ger-Inf-05 study G: effect of low doses of corticosteroids in septic shock patients with or without early acute respiratory distress syndrome. Crit Care Med.

[66] Meijvis SC, Hardeman H, Remmelts HH, Heijligenberg R, Rijkers GT, van Velzen-Blad H, Voorn GP, van de Garde EM, Endeman H, Grutters JC (2011). Dexamethasone and length of hospital stay in patients with community-acquired pneumonia: a randomised, double-blind, placebo-controlled trial. Lancet.

[67] Madamsetty VS, Mohammadinejad R, Uzieliene I, Nabavi N, Dehshahri A, Garcia-Couce J, Tavakol S, Moghassemi S, Dadashzadeh A, Makvandi P (2022). Dexamethasone: insights into pharmacological aspects, therapeutic mechanisms, and delivery systems. ACS Biomater Sci Eng.

