# Supplementary material for: A diagnostic model for sepsis-induced acute lung injury using a consensus machine learning approach and its therapeutic implications
Source: J Transl Med. 2023 Sep 12;21:620. doi: 10.1186/s12967-023-04499-4 (PMC10498641; doi:10.1186/s12967-023-04499-4)
Supplement: Supplementary file 1 — Additional file 1: Figure S1. The architecture of stacking model involves multiple base models (Elastic Net, svm, Random Forest and XGBoost), and a meta-model (stacking ensemble model) that combines the predictions in the base models. The meta-model is trained on the predictions made by base models on out-of-sample data. In this study, the training datasets were prepared for the meta-model is via 10-fold cross-validation of base models, where the out-of-fold predictions are used as the basis for the training dataset for the meta-model. Multiple base-models are often complex and diverse, using a diverse range of models (Elastic Net, svm, Random Forest and XGBoost) that make different assumptions about the prediction. The meta-model is often simple which provide a smooth interpretation of the predictions made by the base models. Thus, linear models were used as the meta-model, and the predictions made is a weighted average of the predictions made by the base models. Figure S2. Data processing. A, B PCA analysis and Box plot of expression profiles before batch effect correction. C, D PCA analysis and Box plot of expression profiles after batch effect correction. E t-SNE plot of subjects before batch correction with Combat. F t-SNE plot of subjects after batch correction with Combat. Figure S3. The functional analysis for sepsis-induced ALI. A Biological process. B Cell component. C Molecular function. D KEGG pathway enrichment analysis. Figure S4. Correlation plot of genes remaining after filtering out those with high correlation (Spearman’s >0.5). Figure S5. The DEGs selected by each machine learning methods. A–D Elastic net, svm, random forest and XGBoost were conducted to select features and sort by importance. Figure S6. The expression levels of genes selected by at least two methods. A The expression of selected genes in sepsis and sepsis-induced ALI. B Heatmap showed the selected genes expression in sepsis and sepsis-induced ALI. Figure S7. Individual diagnostic [file 12967_2023_4499_MOESM1_ESM.docx]

**Supplementary Materials**


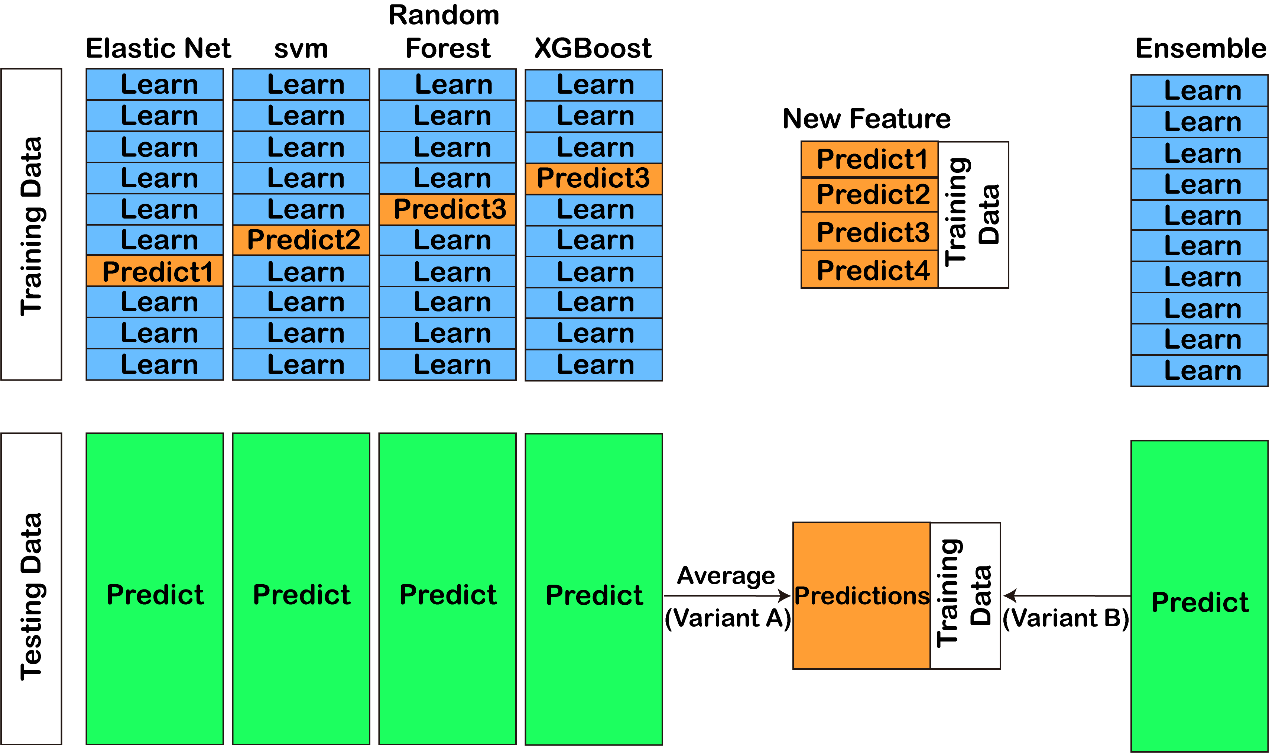


Figure S1. The architecture of stacking model involves multiple base models (Elastic Net, svm, Random Forest and XGBoost), and a meta-model (stacking ensemble model) that combines the predictions in the base models. The meta-model is trained on the predictions made by base models on out-of-sample data. In this study, the training datasets were prepared for the meta-model is via 10-fold cross-validation of base models, where the out-of-fold predictions are used as the basis for the training dataset for the meta-model. Multiple base-models are often complex and diverse, using a diverse range of models (Elastic Net, svm, Random Forest and XGBoost) that make different assumptions about the prediction. The meta-model is often simple which provide a smooth interpretation of the predictions made by the base models. Thus, linear models were used as the meta-model, and the predictions made is a weighted average of the predictions made by the base models.


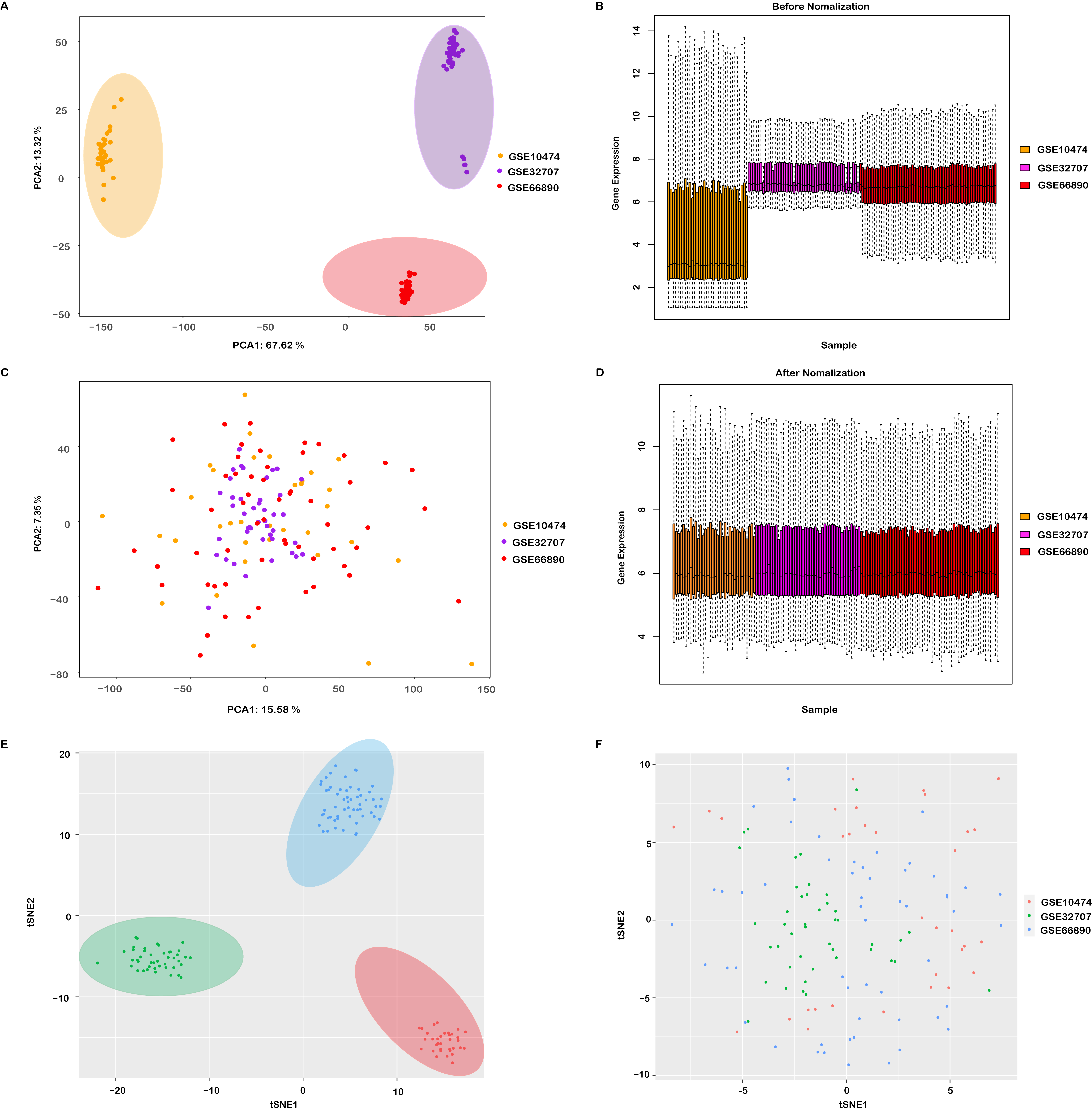


**Figure S2.** Data processing. (A, B) PCA analysis and Box plot of expression profiles before batch effect correction. (C, D) PCA analysis and Box plot of expression profiles after batch effect correction. (E) t-SNE plot of subjects before batch correction with Combat. (F) t-SNE plot of subjects after batch correction with Combat.


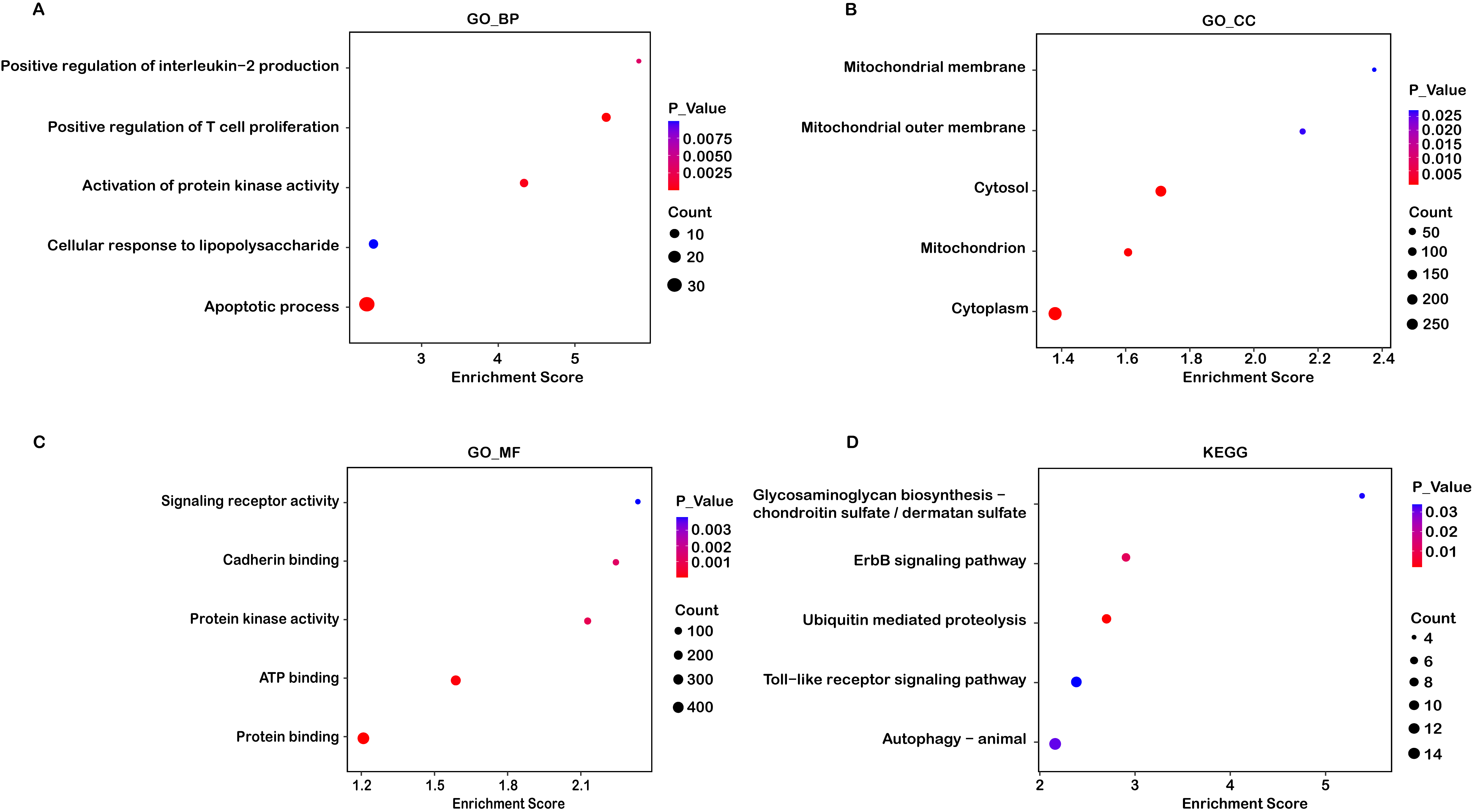


**Figure S3.** The functional analysis for sepsis-induced ALI. (A) Biological process. (B) Cell component. (C) Molecular function. (D) KEGG pathway enrichment analysis.


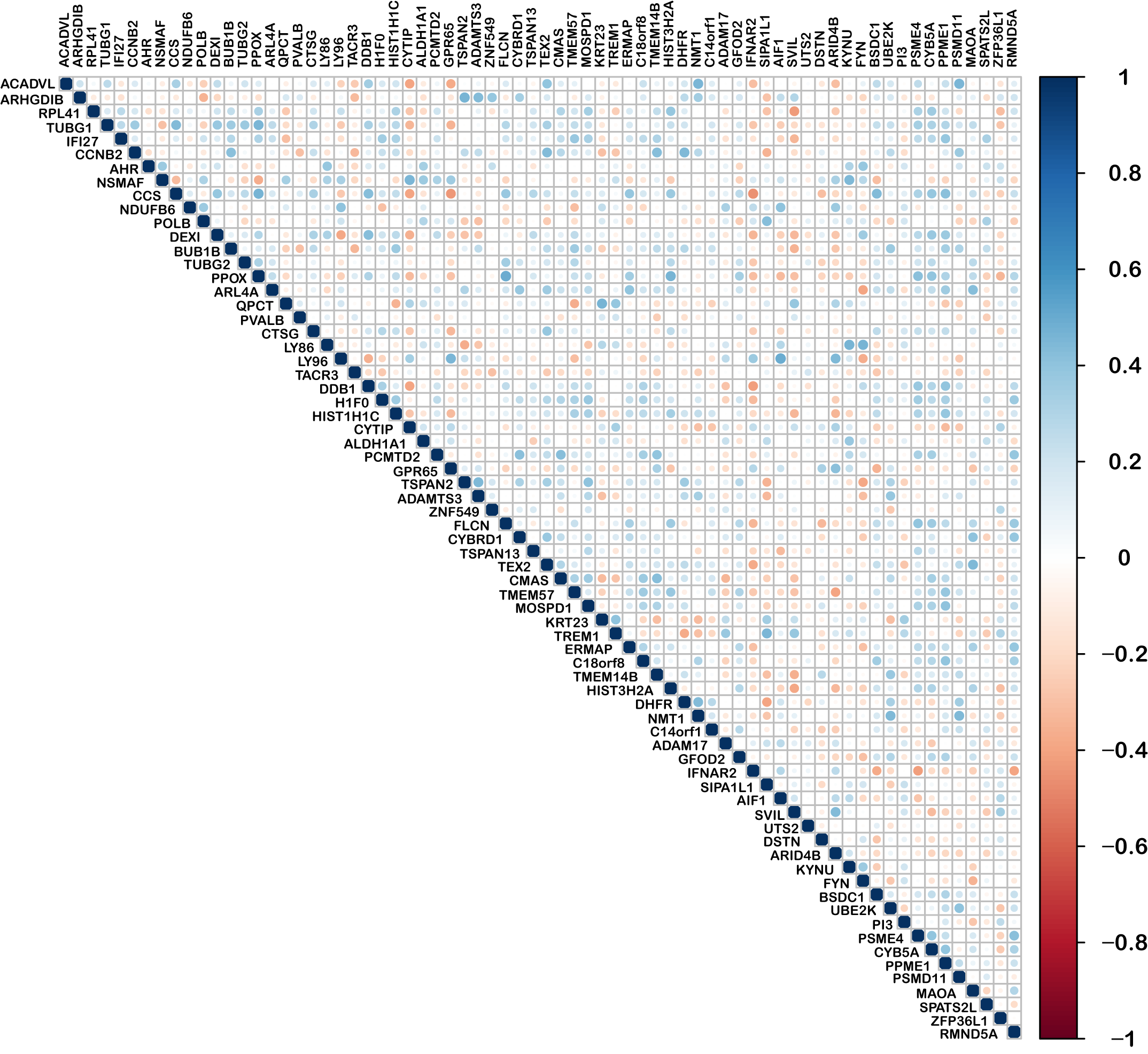


**Figure S4.** Correlation plot of genes remaining after filtering out those with high correlation (Spearman’s >0.5)


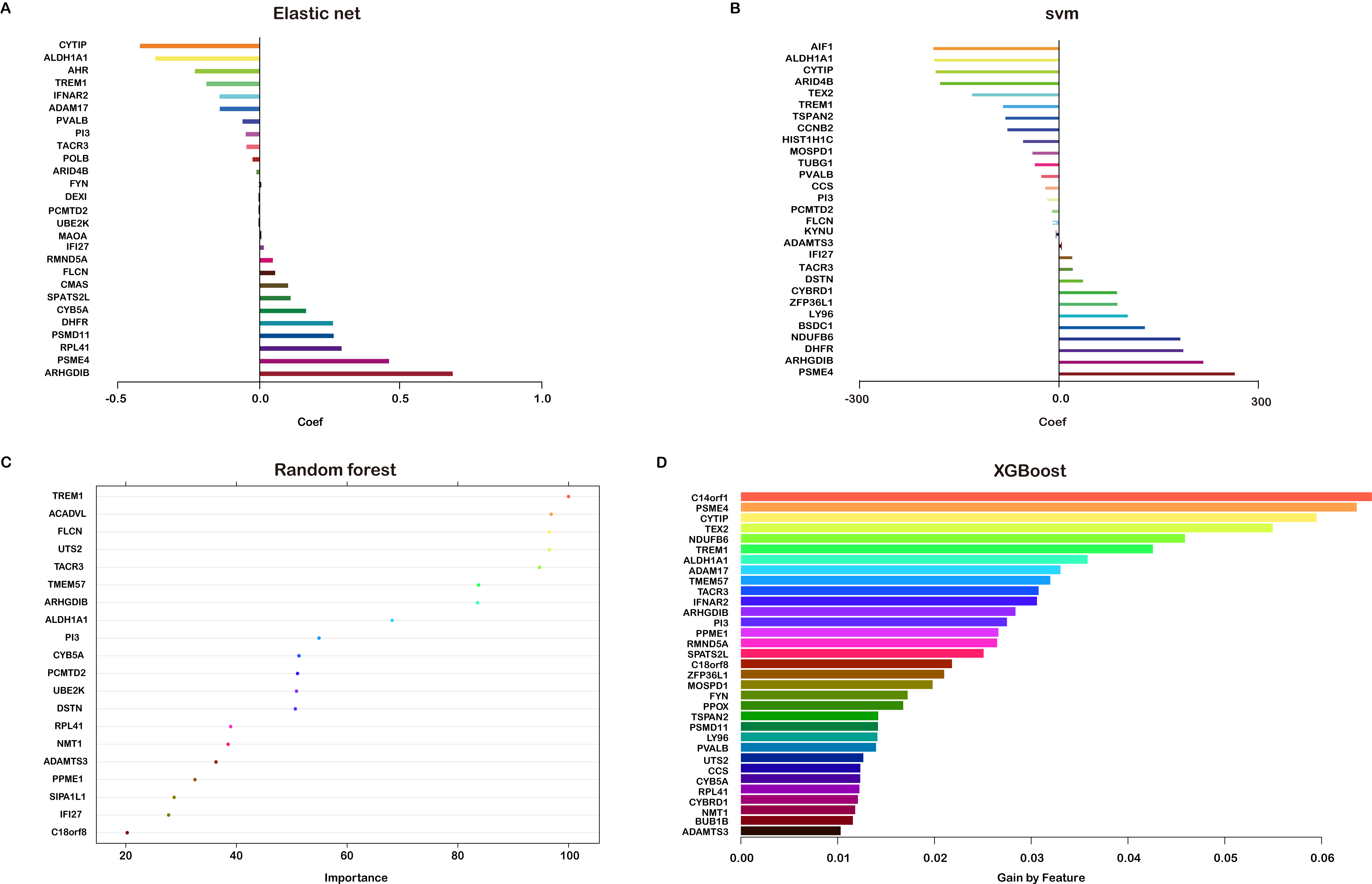


Figure S5. The DEGs selected by each machine learning methods. (A)-(D) Elastic net, svm, random forest and XGBoost were conducted to select features and sort by importance.


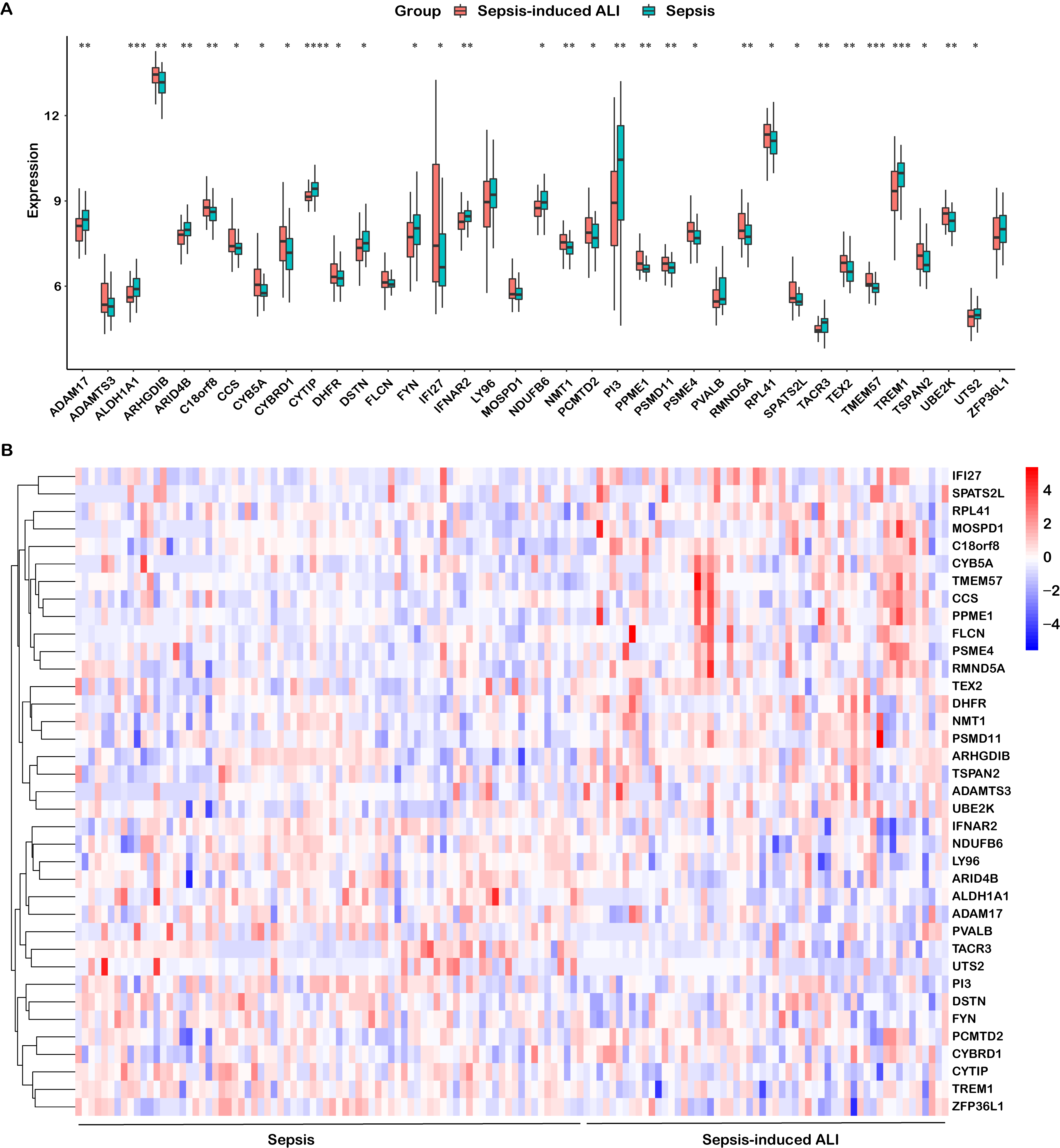


**Figure S6.** The expression levels of genes selected by at least two methods. (A) The expression of selected genes in sepsis and sepsis-induced ALI. (B) Heatmap showed the selected genes expression in sepsis and sepsis-induced ALI.


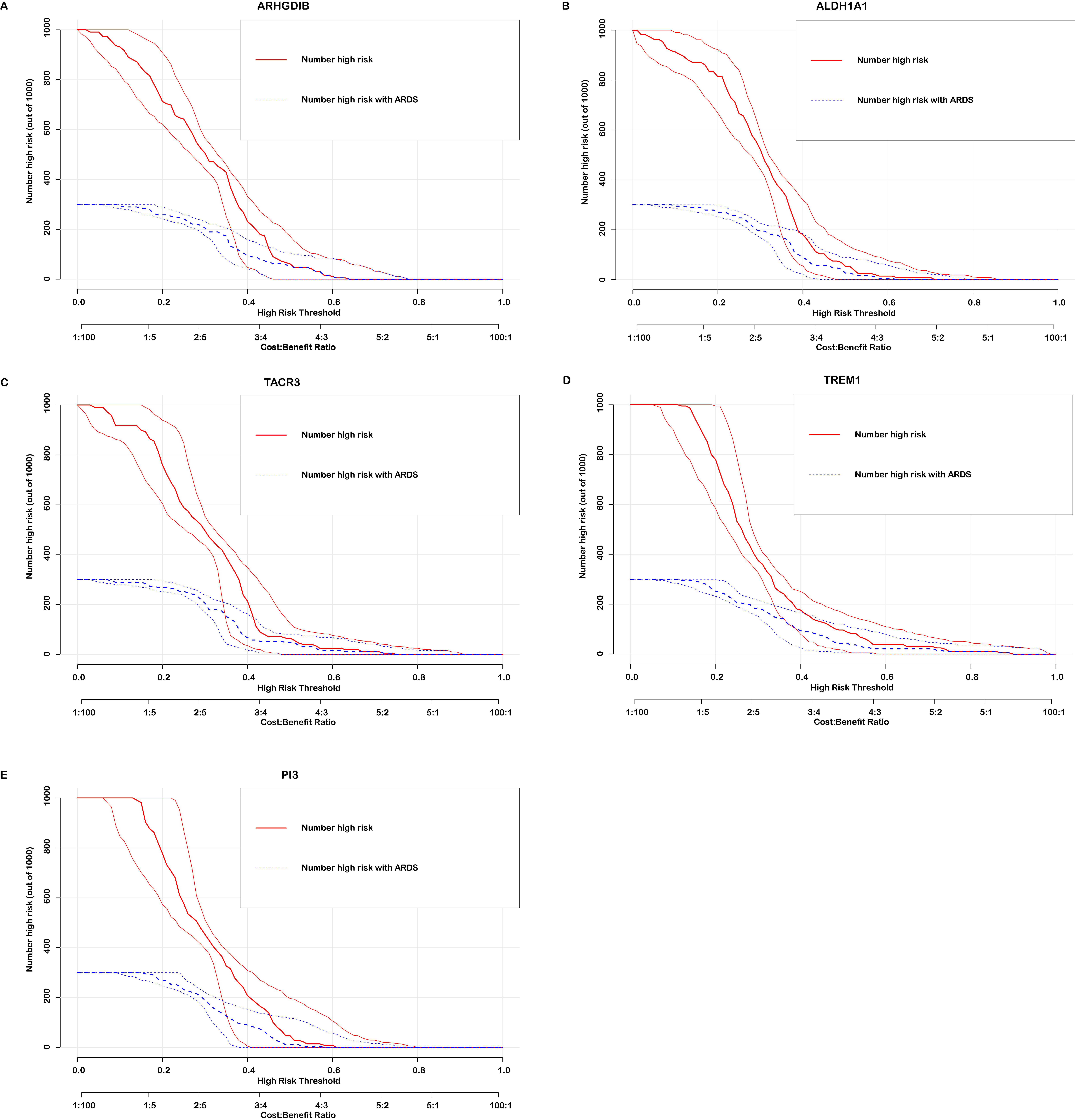


**Figure S7**. Individual diagnostic genes CIC used to assess the performance of nomogram. (A)-(D) The CIC of ARHGDIB, ALDH1A1, TACR3, TREM1 and PI3.


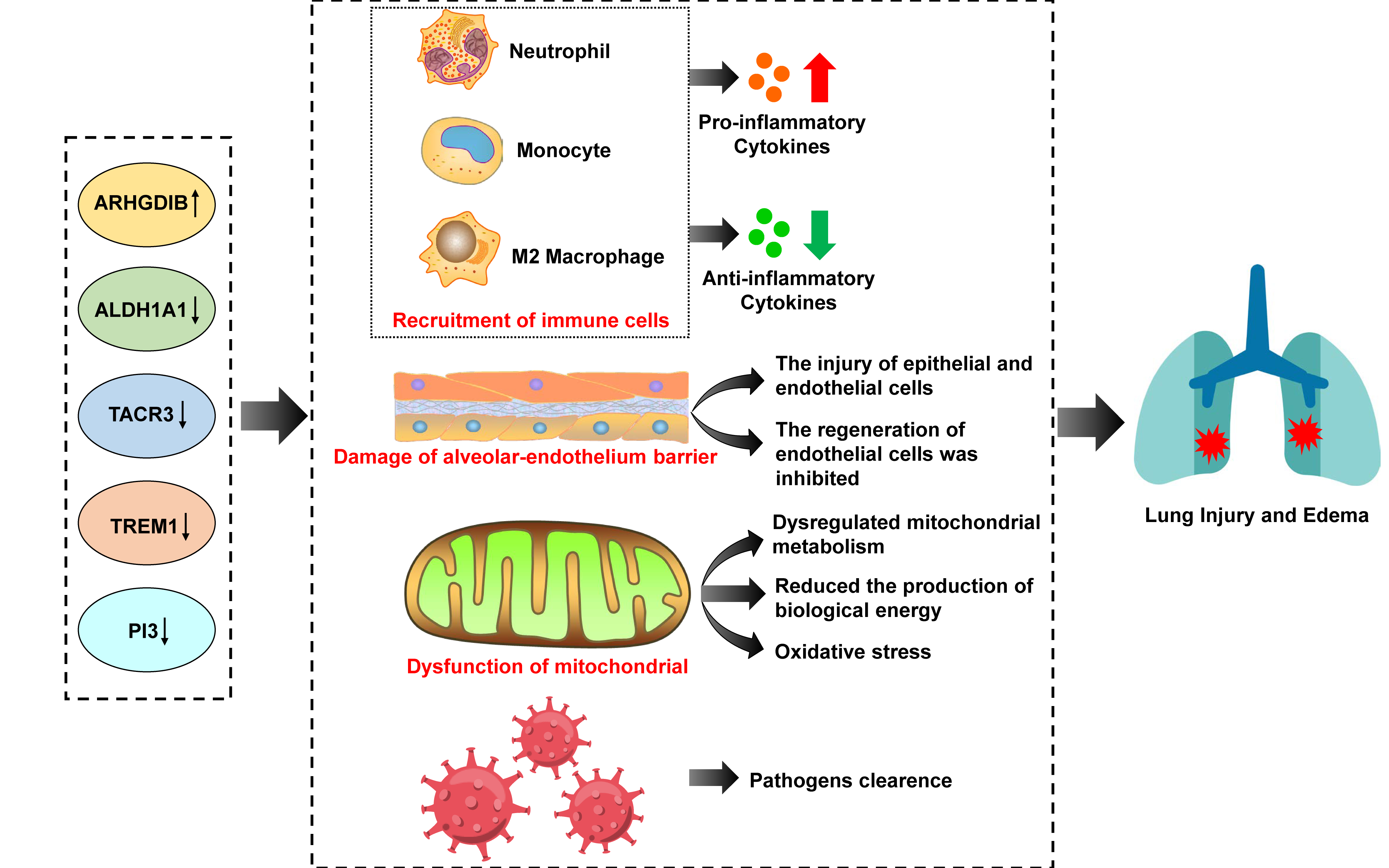


**Figure S8**. Potential mechanisms of diagnostic genes contributing to the development of ALI/ARDS.

**Table S1**. Basic information of the datasets included in this study

| Accession | Cohort description | Timing of gene expression profiling | Sample type | Country | Sepsis-related ALI / Total |
| --- | --- | --- | --- | --- | --- |
| GSE66890 | Sepsis and Sepsis-related ARDS | Within the first 24 hours of admission | Whole blood | United States | 29/57 |
| GSE10474 | Sepsis and Sepsis-related ALI | Within 48 hours of admission | Whole blood | United States | 13/34 |
| GSE32707 | Sepsis and Sepsis-related ARDS | On the day of admission | Whole blood | United States | 18/48 |
| E-MTAB-5273 | Sepsis and Sepsis-related CAP | First day of ICU stay | Circulating leukocytes | UK | 127/221 |
| E-MTAB-5274 | Sepsis and Sepsis-related CAP | First day of ICU stay | Circulating leukocytes | UK | 24/108 |

**Table S2.** Tripod Checklist

| Section/Topic | | | Checklist Item | Section |
| --- | --- | --- | --- | --- |
| Title and abstract | | | | |
| Title | 1 | D; V | Identify the study as developing and/or validating a multivariable prediction model, the target population, and the outcome to be predicted. | Title |
| Abstract | 2 | D; V | Provide a summary of objectives, study design, setting, participants, sample size, predictors, outcome, statistical analysis, results, and conclusions. | Abstract |
| Introduction | | | | |
| Background and objectives | 3a | D; V | Explain the medical context (including whether diagnostic or prognostic) and rationale for developing or validating the multivariable prediction model, including references to existing models. | Introduction |
|  | 3b | D; V | Specify the objectives, including whether the study describes the development or validation of the model or both. | Introduction |
| Methods | | | | |
| Source of data | 4a | D; V | Describe the study design or source of data (e.g., randomized trial, cohort, or registry data), separately for the development and validation data sets, if applicable. | Data sources used for analysis, Table S1. |
|  | 4b | D; V | Specify the key study dates, including start of accrual; end of accrual; and, if applicable, end of follow-up. | Data sources used for analysis, Table S1. |
| Participants | 5a | D; V | Specify key elements of the study setting (e.g., primary care, secondary care, general population) including number and location of centers. | Data sources used for analysis, Table S1. |
|  | 5b | D; V | Describe eligibility criteria for participants. | Data sources used for analysis |
|  | 5c | D; V | Give details of treatments received, if relevant. | n/a |
| Outcomes | 6a | D; V | Clearly define the outcome that is predicted by the prediction model, including how and when assessed. | Multivariable DEGs selection and model building |
|  | 6b | D; V | Report any actions to blind assessment of the outcome to be predicted. | n/a |
| Predictors | 7a | D; V | Clearly define all predictors used in developing or validating the multivariable prediction model, including how and when they were measured. | Data preprocessing and identification of differentially expressed genes (DEGs) |
|  | 7b | D; V | Report any actions to blind assessment of predictors for the outcome and other predictors. | n/a |
| Sample size | 8 | D; V | Explain how the study size was arrived at. | Data sources used for analysis, Table S1. |
| Missing data | 9 | D; V | Describe how missing data were handled (e.g., complete-case analysis, single imputation, multiple imputation) with details of any imputation method. | Data preprocessing and identification of differentially expressed genes (DEGs) |
| Statistical analysis methods | 10a | D | Describe how predictors were handled in the analyses. | Data preprocessing and identification of differentially expressed genes (DEGs); Multivariable DEGs selection and model building |
|  | 10b | D | Specify type of model, all model-building procedures (including any predictor selection), and method for internal validation. | Multivariable DEGs selection and model building; Multivariable classifier performance assessment and validation |
|  | 10c | V | For validation, describe how the predictions were calculated. | Multivariable DEGs selection and model building; Multivariable classifier performance assessment and validation |
|  | 10d | D; V | Specify all measures used to assess model performance and, if relevant, to compare multiple models. | Multivariable classifier performance assessment and validation |
|  | 10e | V | Describe any model updating (e.g., recalibration) arising from the validation, if done. | n/a |
| Risk groups | 11 | D; V | Provide details on how risk groups were created, if done. | n/a |
| Development vs. validation | 12 | V | For validation, identify any differences from the development data in setting, eligibility criteria, outcome, and predictors. | Data sources used for analysis; Table S1. |
| Results | | | | |
| Participants | 13a | D; V | Describe the flow of participants through the study, including the number of participants with and without the outcome and, if applicable, a summary of the follow-up time. A diagram may be helpful. | Data sources used for analysis; Table S1; Figure 1. |
|  | 13b | D; V | Describe the characteristics of the participants (basic demographics, clinical features, available predictors), including the number of participants with missing data for predictors and outcome. | Table S1 |
|  | 13c | V | For validation, show a comparison with the development data of the distribution of important variables (demographics, predictors and outcome). | Table S1 |
| Model development | 14a | D | Specify the number of participants and outcome events in each analysis. | Table S1 |
|  | 14b | D | If done, report the unadjusted association between each candidate predictor and outcome. | n/a |
| Model specification | 15a | D | Present the full prediction model to allow predictions for individuals (i.e., all regression coefficients, and model intercept or baseline survival at a given time point). | Multivariable DEGs selection and model building; Multivariable classifier performance assessment and validation; Table S2. |
|  | 15b | D | Explain how to use the prediction model. | Multivariable DEGs selection and model building |
| Model performance | 16 | D; V | Report performance measures (with CIs) for the prediction model. | Table 1 |
| Model updating | 17 | V | If done, report the results from any model updating (i.e., model specification, model performance). | n/a |
| Discussion | | | | |
| Limitations | 18 | D; V | Discuss any limitations of the study (such as nonrepresentative sample, few events per predictor, missing data). | Discussion |
| Interpretation | 19a | V | For validation, discuss the results with reference to performance in the development data, and any other validation data. | Discussion |
|  | 19b | D; V | Give an overall interpretation of the results, considering objectives, limitations, results from similar studies, and other relevant evidence. | Discussion |
| Implications | 20 | D; V | Discuss the potential clinical use of the model and implications for future research. | Discussion |
| Other information | | | | |
| Supplementary information | 21 | D; V | Provide information about the availability of supplementary resources, such as study protocol, Web calculator, and datasets. | Supplementary Information; Availability of data and materials |
| Funding | 22 | D; V | Give the source of funding and the role of the funders for the present study. | Funding |

**Table S3**: Parameters used to optimise an XGBoost classifier for sepsis-induced using genes. a: the range of each parameter tuned, b: the optimal parameter for the initial xgboost model, c: the final parameter value used for an xgboost model trained on a reduced number of genes.

| **Parameter** | **Available Range** | **Optimisation range**^a^ | **Initial value**^b^ | **Optimal value**^c^ |
| --- | --- | --- | --- | --- |
| No of trees | 1 - ∞ | 100 - 10 000 | 4300 | 200 |
| Learning rate | 0 - 1 | 0∙01, 0∙025, 0∙05, 0∙1, 0∙2, 0∙3 | 0∙025 | 0∙025 |
| Maximum tree depth | 0 - ∞ | 1, 2, 3, 4, 5, 6 | 1 | 3 |
| gamma | 0 - ∞ | 0, 0∙05, 0∙1, 0∙5, 0∙7, 0∙9, 1 | 0∙05 | 0.5 |
| Minimum child weight | 0 - ∞ | 1,2,3,4 | 2 | 2 |
| Subsample rate (row sampling) | 0 - 1 | 0∙5, 0∙75, 1∙0 | 0∙5 | 0∙5 |
| % feature used in each boost (column sampling) | 0 - 1 | 0∙4, 0∙6, 0∙8, 1∙0 | 0∙4 | 1 |
